# Differential Profile of Systemic Extracellular Vesicles From Sporadic and Familial Alzheimer’s Disease Leads to Neuroglial and Endothelial Cell Degeneration

**DOI:** 10.3389/fnagi.2020.587989

**Published:** 2020-11-11

**Authors:** Juan Villar-Vesga, Julián Henao-Restrepo, Daniëlle C. Voshart, David Aguillon, Andrés Villegas, Diana Castaño, Julián D. Arias-Londoño, Inge S. Zuhorn, Laís Ribovski, Lara Barazzuol, Gloria P. Cardona-Gómez, Rafael Posada-Duque

**Affiliations:** ^1^Neuroscience Group of Antioquia, Cellular and Molecular Neurobiology Area, Faculty of Medicine, Sede de Investigación Universitaria, University of Antioquia, Medellín, Colombia; ^2^Institute of Biology, Faculty of Exact and Natural Sciences, University of Antioquia, Medellín, Colombia; ^3^Department of Radiation Oncology, University Medical Center Groningen, University of Groningen, Groningen, Netherlands; ^4^Section of Molecular Cell Biology, Department of Biomedical Sciences of Cells and Systems, University Medical Center Groningen, University of Groningen, Groningen, Netherlands; ^5^Neurobank, Neuroscience Group of Antioquia, Faculty of Medicine, Sede de Investigación Universitaria, University of Antioquia, Medellín, Colombia; ^6^Grupo de Inmunología Celular e Inmunogenética, Instituto de Investigaciones Médicas, Facultad de Medicina, Universidad de Antioquia, Medellín, Colombia; ^7^Department of Systems Engineering, University of Antioquia, Medellín, Colombia; ^8^Department of Biomedical Engineering, University Medical Center Groningen, University of Groningen, Groningen, Netherlands

**Keywords:** Alzheimer’s disease, extracellular vesicle, proteomic analyses, platelet, endothelium, organoids, leukocyte, astrocycte

## Abstract

Evidence suggests that extracellular vesicles (EVs) act as mediators and biomarkers of neurodegenerative diseases. Two distinct forms of Alzheimer disease (AD) are known: a late-onset sporadic form (SAD) and an early-onset familial form (FAD). Recently, neurovascular dysfunction and altered systemic immunological components have been linked to AD neurodegeneration. Therefore, we characterized systemic-EVs from postmortem SAD and FAD patients and evaluated their effects on neuroglial and endothelial cells. We found increase CLN-5 spots with vesicular morphology in the abluminal portion of vessels from SAD patients. Both forms of AD were associated with larger and more numerous systemic EVs. Specifically, SAD patients showed an increase in endothelial- and leukocyte-derived EVs containing mitochondria; in contrast, FAD patients showed an increase in platelet-derived EVs. We detected a differential protein composition for SAD- and FAD-EVs associated with the coagulation cascade, inflammation, and lipid-carbohydrate metabolism. Using mono- and cocultures (endothelium-astrocytes-neurons) and human cortical organoids, we showed that AD-EVs induced cytotoxicity. Both forms of AD featured decreased neuronal branches area and astrocytic hyperreactivity, but SAD-EVs led to greater endothelial detrimental effects than FAD-EVs. In addition, FAD- and SAD-EVs affected calcium dynamics in a cortical organoid model. Our findings indicate that the phenotype of systemic AD-EVs is differentially defined by the etiopathology of the disease (SAD or FAD), which results in a differential alteration of the NVU cells implied in neurodegeneration.

## Highlights

- Alzheimer’s disease (AD) enhances the number of released brain microvascular and systemic extracellular vesicles (EVs).
- Surface markers of systemic EVs are associated with the leukocyte-endothelium in sporadic Alzheimer’s disease (SAD) and with platelets in familial Alzheimer’s disease (FAD).
- Protein composition in AD-EVs are implicated in the coagulation cascade (fibrinogen), the complement cascade, inflammation and lipid-carbohydrate metabolism.
- The expression of SORD, SAA4, SERPINF1, PLTP, APOE, APOA2, IGKV1D-33, IGKV1-33 and C8A distinguishes control-, SAD- and FAD-EVs.
- The expression of PEBP1, CFD, and C6 is a signature of SAD-EVs, while the expression of SORD, PLTP, and IGGKV1D-33 is a signature of FAD-EVs.
- AD-EVs induce endothelial disruption, astrocyte hyperactivation, neuronal death, and calcium dysregulation.
- The detrimental effects on the vasculature caused by SAD-EVs are higher than those caused by FAD-EVs.

## Introduction

Alzheimer’s disease (AD) is the primary cause of dementia (Ferri et al., 2005) and is a growing public health issue worldwide (Prince et al., 2016). No treatments to cure or halt the progression of AD are currently available, and validated systemic biomarkers for AD diagnosis have also not been established. AD is a heterogenic and chronic disease (Querfurth and LaFerla, 2010) that is characterized by amyloidosis, tauopathy and neurodegeneration (Jack et al., 2016). However, neurovascular unit (NVU) degeneration has also been proposed (Zlokovic, 2011), which includes neuronal death, astrogliosis, microgliosis, and blood–brain barrier (BBB) damage (De Strooper and Karran, 2016) as well as calcium dysregulation (LaFerla, 2002; Jadiya et al., 2019), oxidative stress and mitochondrial dysfunction (Burté et al., 2015). Neuroinflammation has recently been implicated in AD and includes microgliosis, leukocyte infiltration in the brain and proposed systemic immune component alterations (Morris et al., 2019). However, it is unclear which molecular and cellular mediators drive AD. A recent hypothesis points to neuroimmune activation, where systemic and BBB damage dysregulate the coagulation process and activate complement and platelets (De Luca et al., 2018). These events lead to infiltration of the parenchyma by leukocytes and parenchymal brain damage (Ramirez et al., 2018; Passamonti et al., 2019). Additionally, recent studies have suggested that EVs may be important mediators of AD (Goetzl et al., 2016; Dickhout and Koenen, 2018; Gallart-Palau et al., 2019). Brain-derived and systemic EVs could mediate systemic inflammation as well as brain and NVU dysfunction.

Extracellular vesicles are a heterogeneous group of vesicle structures delimited by a lipid bilayer that are produced by the processes of budding and exocytosis; as a result, EVs contain cytosolic and membrane components of their parent cells (Van Niel et al., 2018). EVs have the ability to exchange components between cells (Joshi et al., 2020), which allows the transfer of nucleic acids, lipids, proteins (functional enzymes) and organelles (mitochondria); therefore, EVs act as intercellular communicators and signaling vehicles in homeostatic and pathological processes (Yáñez-Mó et al., 2015; Van Niel et al., 2018). EVs altered in terms of number or phenotype are released upon cell activation or injury, which initiates signaling to recipient cells (Van Niel et al., 2018). Growing evidence indicates that EVs are important in a variety of chronic inflammatory (Schindler et al., 2014; Burbano et al., 2015; Malm et al., 2016), neurodegenerative and neuroinflammatory diseases (Malm et al., 2016; Li T.R. et al., 2019). In the latter, EVs can favor the spread of protein aggregates (Pérez et al., 2019), induce leukocyte infiltration to the brain parenchyma and carry proinflammatory messengers (Ramirez et al., 2018; Yang et al., 2018). Interestingly, in neuroinflammation, EVs serve as vehicles that transfer claudin-5 (CLN-5), a major tight junction protein, at sites of leukocyte-endothelial contact along the BBB, which induces leukocyte infiltration (Paul et al., 2016).

Systemic EVs can be released from different cell sources, such as platelets, leukocytes, erythrocytes and endothelial cells (Akbar et al., 2019). Activation of these cell types during neuroimmune activation (De Luca et al., 2018) could affect the profile of systemic EVs. We propose EVs as systemic components that may be associated with AD. In AD, EVs have been proposed to be biomarkers and tracers of the disease (Kapogiannis et al., 2019). Systemic neuronal-derived EVs from AD brains exhibited downregulated mi-RNA (Cha et al., 2019), were increased in number and diameter and a differential protein content (Pulliam et al., 2019). Moreover, endothelial-derived EVs have been found to be increased in AD patients (Hosseinzadeh et al., 2018), which could reflect BBB dysfunction. Moreover, we propose that altered systemic EVs in AD could transfer a variety of proteins that affect NVU homeostasis via the induction of BBB dysfunction and neurotoxicity (Laske et al., 2015; Yang et al., 2018).

The vast majority of AD cases occur sporadically and are related to environmental risk factors, lifestyle and genetic risk factors; this type of AD is known as sporadic Alzheimer’s disease (SAD) and is manifested in elderly individuals (Lane et al., 2018; Sims et al., 2020). However, some mutations cause a rare form of AD called familial AD (FAD) in which patients develop symptoms at an earlier age (Bateman et al., 2011). Notably, Antioquia has the largest and most homogeneous family group with FAD reported to date; this family has the “paisa” *E280A* mutation in the Presenilin-1 (*PS1*) gene (Lopera et al., 2013). Previous work by our group identified a differential lipid profile in the brains of SAD and FAD patients (Villamil-Ortiz et al., 2018), which was found to be related to lipid mitochondrial alterations in AD. Using a similar approach, we studied these two AD forms as well as the association between the systemic EV profile and NVU degeneration.

Thereafter, studies of the systemic EV profile have provided crucial information about systemic alterations, the inflammatory state, changes in the BBB and NVU degeneration in patients with AD. Understanding the importance of systemic-EVs in AD suggest their potential role as prodromal biomarkers that could be used to clarify some early events in AD pathogenesis and that they could serve as a basis for directed future preventive treatment. The aim of our study was to characterize systemic EVs from SAD and FAD patients and to evaluate their effects on neuroglial and endothelial cells using mono- and cocultures as well as human cortical brain organoids. The profile and proteome of the EV surface were analyzed to propose a differential mechanism associated with these two types of AD.

## Materials and Methods

### Human Samples

This study was approved by the bioethical committee for human studies at the University of Antioquia, and all Alzheimer’s disease (AD) patients and healthy controls provided informed consent for research and sample use. We included overall postmortem blood or brain samples from 10 control cases, 6 cases of SAD, and 10 cases of FAD (PS1 mutation E280A) obtained from the University of Antioquia’s biobank. We included the following sample data: postmortem index, which was the time that elapsed between the patient’s death and sampling; Braak stage to classify the degree of AD; the score from the Consortium to Establish a Registry for Alzheimer’s Disease (CERAD); Thal phasing scale of senile plaque accumulation and The National Institute on Aging—Alzheimer’s Association (NIA-AA) Alzheimer’s Diagnostic Framework, to characterize the pathological diagnosis of AD as “high likelihood”; (Table 1), which is a neuropsychological battery developed to screen AD; and comorbidities listed in Table 1.

**TABLE 1:** Description of postmortem samples.

Case	Sex	Age of onset	Age of death	P.I. (*h*)	CERAD	Braak	Thal	NIA-AA	Comorbidities
Control	F	NAP	84	4.3	B	1	2	A1, B1, C2	Hypertension, type II diabetes mellitus, pancreatitis
Control	F	NAP	67	3.8	0	1	0	A0, B1, C0	Hypertension, hypothyroidism
Control	F	NAP	75	14.8	0	0	0	A0, B0, C0	Hypertension
Control	M	NAP	69	6.8	A	1	2	A1, B1, C1	Hypertension, chronic venous insufficiency
Control	M	NAP	61	5.3	0	0	0	A0, B0, C0	Hypertension, type II diabetes mellitus
Control	M	NAP	46	6	0	1	0	A0, B1, C0	None
Control	M	NAP	84	3	0	3	0	A0, B2, C0	Hypertension, chronic renal insufficiency, chronic obstructive lung disease
Control	F	NAP	44	4.4	0	0	0	A0, B0, C0	Depression
Control	M	NAP	38	3.4	0	0	0	A0, B0, C0	None
Control	M	NAP	73	3.5	B	1	2	A1, B1, C2	None
SAD	M	52	68	5.1	B	5	5	A3, B3, C2	Hypertension, dyslipidemia, coronary heart disease, alcoholism
SAD	F	75	85	3.3	B	5	5	A3, B3, C2	Hypertension, hypothyroidism, congestive heart failure
SAD	F	81	94	4	B	4	5	A3, B2, C2	Hypertension, type II diabetes mellitus, breast cancer
SAD	F	79	90	3.3	C	4	4	A3, B2, C3	Hypertension, dyslipidemia
SAD	F	59	73	3.5	C	5	4	A3, B3, C3	Dyslipidemia, depression
SAD	F	92	98	4.3	A	3	3	A2, B2, C1	Hypertension
FAD (E280A)	F	44	50	5.2	C	6	5	A3, B3, C3	None
FAD (E280A)	M	46	62	4.3	C	6	5	A3, B3, C3	Hypertension, alcoholism, traumatic brain injury
FAD (E280A)	F	50	63	4.9	C	6	5	A3, B3, C3	Hypothyroidism, chronic venous insufficiency
FAD (E280A)	M	49	59	5	C	6	5	A3, B3, C3	Dyslipidemia, alcoholism
FAD (E280A)	F	51	65	3.8	B	5	5	A3, B3, C2	Type II diabetes mellitus, dyslipidemia, hypothyroidism
FAD (E280A)	M	43	49	11.9	C	5	5	A3, B3, C3	Congenital valvular disease, hypothyroidism
FAD (E280A)	M	42	53	2.8	C	6	5	A3, B3, C3	None
FAD (E280A)	M	58	69	5.1	B	5	5	A3, B3, C2	Hypertension
FAD (E280A)	F	41	56	5.0	C	5	5	A3, B3, C3	Hypertension
FAD (E280A)	M	44	57	1.8	C	5	5	A3, B3, C3	Traumatic brain injury

*Clinical data of patients used for the different experimental procedures. 0, no histological evidence of Alzheimer’s disease; A, histological findings provide UNCERTAIN evidence of Alzheimer’s disease; B, histological findings SUGGEST the diagnosis of Alzheimer’s disease; C, histologic findings indicate the diagnosis of Alzheimer’s disease. F, female; M, male; NAP, not applicable; P.I., Postmortem Index.*

Each patient sample was considered as independent *n*. To perform the different experimental approaches, we included the following sample sizes: brain samples from CNT, *n* = 5; SAD, *n* = 5; and FAD, *n* = 7 for immunohistochemistry and immunofluorescence; blood samples from CNT, *n* = 6; SAD, *n* = 6; FAD, *n* = 6 for flow cytometry analysis; blood samples from CNT, *n* = 6; SAD, *n* = 5; FAD, *n* = 6 for nanotracking analysis and cortical brain organoid stimuli; blood samples from CNT, *n* = 5; SAD, *n* = 5; FAD, *n* = 5 for proteomic analysis and cell stimuli; CSF samples from *n* = 4; SAD, *n* = 5; FAD, *n* = 5 for flow cytometry counting; and blood samples from CNT, *n* = 3; SAD, *n* = 3; FAD, *n* = 3 for western blotting, transcytosis and organoid pool stimuli.

### Immunohistochemistry and Immunofluorescence

**FIGURE 1:**
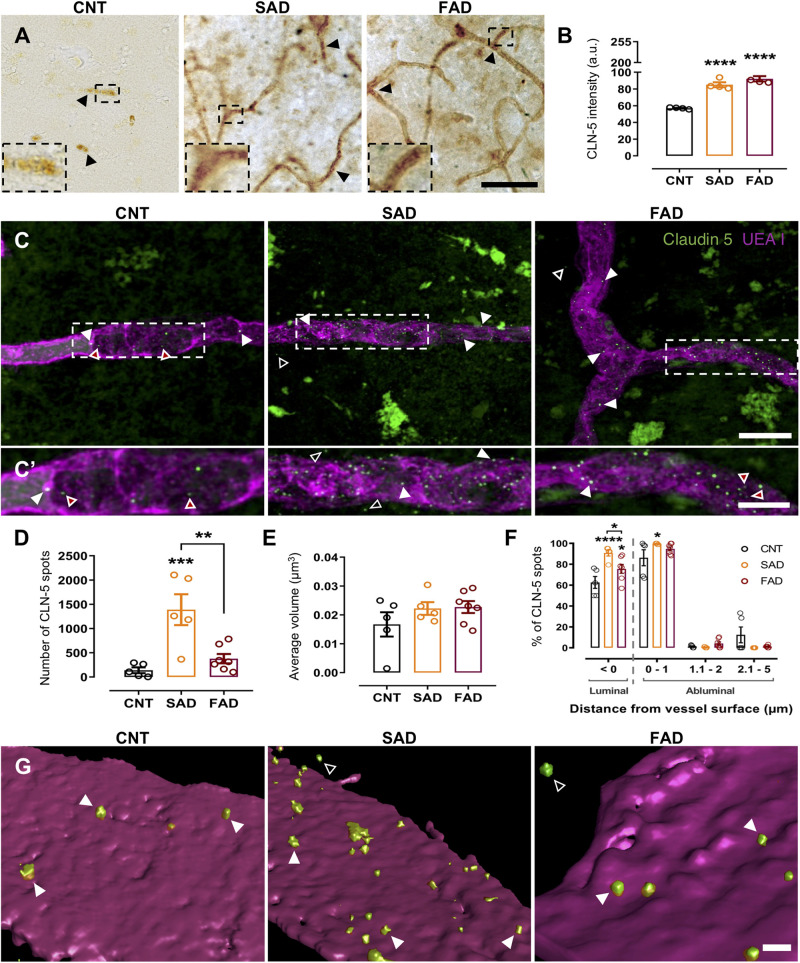
CLN-5+ spots are increased in the brain microvasculature of AD patients. CLN-5 immunohistochemistry analysis in the human brain microvasculature: **(A)** Representative images of CLN-5 reactivity show increased expression and a dotted-like staining pattern in AD (insets). Quantification of the CLN-5 intensity is shown in panel **(B)**. Confocal image analysis of the human brain microvasculature: **(C)** Z projections of the labeled brain microvasculature showing CLN-5+ spots near the vessel wall in CNT, SAD, and FAD tissues. **(C’)** 2× magnification of the insets in panel **(C)**. **(D,E)** Analysis of the CLN-5 signal revealed **(D)** the total number of CLN-5 spots present inside and from 0 to 5 μm away from the vessel wall and **(E)** their volumes. **(F)** Percentage of the total CLN-5+ spots present luminally (left panel) and percentage distribution of the abluminal CLN-5+ spots at different distances from the vessel surface (right panel). **(G)** 3D reconstruction showing CLN-5+ spots either on or away from the UEA I-stained endothelial basement membrane in CNT, SAD, and FAD tissues. **(A)** The CLN-5 microvascular dotted-like staining pattern is indicated by black arrowheads. **(B)** Intensity values are presented as arbitrary units (a.u.) from the mean gray value which ranges from 0 to 255. **(C,C’,G)** Luminal, abluminal and directly apposed CLN-5+ spots on the vessel surface are indicated by red-filled, empty and white arrowheads, respectively. Scale bars: **(A)** 50 μm; **(C)**, 10 μm; **(C’)**, 5 μm; **(G)**, 1 μm. Representative data from CNT, *n* = 5; SAD, *n* = 5; FAD, *n* = 7. Data are plotted as means and SEM. One-way ANOVA, Tukey’s multiple comparison test. * indicates *p* < 0.05; ** indicates *p* < 0.01; *** indicates *p* < 0.001; **** indicates *p* < 0.0001.

Cortical samples from the middle frontal gyrus were collected and immediately fixed in 4% paraformaldehyde prepared in cytoskeleton buffer (Posada-Duque et al., 2013) for 72 h at 4°C; the solution was replaced every 24 h. These cortical fragments were sectioned into coronal slices 50 μm thick using a vibratome (Leica, VT1000 S). Antigen retrieval was performed by exposing the tissue to 98% formic acid at 85°C for 5 min. For the immunohistochemistry (IHC) assay, endogenous peroxidase activity was blocked by incubation in 1:1 methanol-hydrogen peroxide at a 1% concentration in 0.1 M phosphate buffer (PB). In the case of immunofluorescence (IF), autofluorescence was blocked using 50 mM NH_4_Cl. To avoid non-specific binding by the antibodies, the samples were incubated in 1% bovine serum albumin (BSA; Sigma-Aldrich, Cat. # A9647) for 1 h, followed by incubation with a primary mouse claudin 5 antibody (Invitrogen; Cat. #35-2500; 1:500) diluted in 0.3% BSA, 0.3% Triton X and 1 M PB (pH 7.4) for 72 h at 4°C. Excess antibody was washed away, and the corresponding secondary antibodies– a biotinylated goat anti-mouse antibody (Invitrogen; Cat. #31800; 1:250) for IHC or a goat anti-mouse Alexa Fluor^®^ 488 antibody (Invitrogen; Cat. #A-11001; 1:750) and the endothelial basement membrane stain DyLight 649 lectin UEA I (Vector Labs; Cat. #DL-1068; 1:500) for IF– were applied for 1 h at room temperature. In the case of IHC, the previous step was followed by incubation with avidin-biotin peroxidase complex (Thermo Scientific; Cat. #32020; 1:250) for 1 h and the chromogen 3,3′-diaminobenzidine for 5 min. The sections were repeatedly washed, sequentially dehydrated in ethanol 70%, 96%, 100% and xylene and finally mounted on glass slides with Shandon Consul-Mount (Thermo Scientific; Cat. #9990440). Four complete samples (∼3 cm^3^ from each tissue) representing each experimental condition were captured by bright-field microscopy using an Olympus CX35 equipped with a Nikon DS-5M camera (Figure 1A and Supplementary Figures S1A,B). All the mean gray values were measured in FIJI (NIH ImageJ software) and expressed as arbitrary units (a.u.). In the case of IF, the slides were mounted hydrated with FluorSave Reagent (Millipore; Cat. #345789), the capture protocol for which is described in the next section.

### Confocal Imaging

Three high magnification images of the third layer of gray matter were acquired per slide using a Perkin Elmer Ultraview RS Spinning Disk Confocal Microscope equipped with a 12−bit CCD camera (Hamamatsu ORCA-ER), a 40× oil-immersion objective (NA 1.3; C−Apochromat; Zeiss) with 1.6 optobar, two diode lasers of 488 and 655 nm (Omicron) and Volocity^®^ 4.2 software (Improvision). Then, 16-bit TIFF images of 1344 × 1024 pixels (139.44 × 106.24 μm) were obtained with an XY pixel size of 103.75 nm and 300 nm between Z−sections. At least 30 optical slices of each field were registered (9 μm thick).

### Image Processing and Quantification Analysis

First, the images were deconvolved using Huygens Professional 19.10 software (Scientific Volume Imaging B.V.). The signal noise ratio (SNR) used for deconvolution was 40 for all images in both channels. Subsequently, 8-bit images were processed and analyzed in FIJI. The UEA I signal was easily segmented using intensity thresholding. To retrieve and quantify CLN-5 signals from the vessel surface, the background was subtracted using a rolling ball radius of 10 pixels, and local contrast was enhanced using a block size of 10. The resulting images were binarized by filtering out intensities below the remaining background mean value. Definitive segmentation of the CLN-5 spots was performed using the Find Connected Regions plugin. Particles exceeding the size of the desired signals (60 voxels) were excluded using the 3D Objects Counter tool. On the contrary, regions of interest (ROIs) describing the segmented microvessels and expanded ROIs at a distance of 5 μm surrounding the 3D microvessel surface were delineated from the UEA I binary images. Segmented CLN-5 spots present inside these ROIs were counted and their volumes were measured using the 3D Objects Counter tool. The distance of the CLN-5 spots from the UEA I- labeled surface was determined using the 3D Distance Map tool from the 3D Suite plugin. This procedure allowed us to discriminate the luminal vesicular particles (CLN-5 spots localized to <0 μm from UEA I-label) and luminal vesicular particles (CLN-5 spots localized to 0–5 μm from UEA I-label). Z projections of the deconvolved images (using the Sum Slices function for UEA I stacks and the standard deviation for CLN-5) and 3D reconstructions of the segmented images were created in FIJI and Huygens Essential 19.10 software, respectively, for illustrative purposes.

### Isolation of EVs

Extracellular vesicles were isolated from citrate-induced anticoagulated peripheral blood and cerebrospinal fluid (CSF), as previously was described in our recent investigations (Burbano et al., 2018a,b, 2019; Villar-Vesga et al., 2019). Briefly, blood samples were centrifuged immediately after collection at 1,800 × *g* for 10 min at 21°C to separate the plasma. Plasma and CSF were centrifuged at 3,000 × *g* for 20 min at 21°C to obtain platelet-poor plasma and CSF with no cell debris. Both fractions were then centrifuged again at 16,900 × *g* for 1 h at 21°C to enrich for EVs. The EV pellets were immediately frozen in 100 μL of filtered Dulbecco’s phosphate-buffered saline (DPBS 1X, GibcoBRL) at −70°C until use for characterization or cell stimulation.

### Nanotracking Analysis and Flow Cytometry of EVs

Extracellular vesicles from the different study groups were thawed and processed in parallel on the day of quantification. We analyzed the particle size of systemic-EVs using NanoSight (LM14, NanoSight) Nanotracking analysis. Particles were detected using a CCD camera, and eight fields were recorded per sample; particle motion was recorded in each field at 25 fps for 60 s. NTA software (NanoSight) was used to plot the resulting distribution diagrams. The NTA results included the concentration of particles and size distribution analysis of the mean, D10, D50, and D90 measurements. The EV concentration and size were also evaluated by flow cytometry, as previously described (Burbano et al., 2018b). Briefly, we acquired all events present in 100 μL of plasma or CSF. Using reference polystyrene spheres (0.1, 0.5, 1, 2, 3, and 6 μm), we compared the EV distribution in FSC-A parameters in terms of forward scattering. We acquired samples in LSR Fortessa (Becton Dickinson, BD), after which the number of EVs was analyzed and estimated using FlowJo 7.6.1 software (Burbano et al., 2018b).

### Phenotype of EVs

Systemic EVs were characterized as previously described (Burbano et al., 2018b; Villar-Vesga et al., 2019) by staining human platelets with anti-CD41a FITC (Clone HIP8, Biolegend), human leukocytes with anti-CD45 PE (Clone HI30, BD), human endothelium with anti-CD10 PE (Clone 43A3, Biolegend), human erythrocytes with anti-CD235a V450 (Clone HI264, Biolegend), human astrocytes with anti-AQ4 FITC (Polyclonal, Bioss), and human neuron-like cells with anti-CD90 PE (Clone 5E10, Biolegend) (Nekludov et al., 2017). Phosphatidylserine (PS) exposure was tested using Annexin V PE (Abcam) in Annexin V binding buffer (BD, contains calcium), and mitochondria were detected using a DiOC6 (dihexyloxacarbocyanine iodide) probe (Invitrogen). For each sample or patient, we separated the sample in four equal parts and generated four different combinations of markers adjusted to the available fluorescence wavelengths. Combinations: 1. CD45 (FITC) + CD105 (PE) + CD235a (Pacific Blue); 2. Annexin V (Cy 5.5) + CD41a (FITC); 3. DIOC6 (FITC); 4. CD90 (PE) + AQ4 (FITC). Flow cytometry parameters were compensated for each marker and combination. The staining and acquisition of these samples were performed in parallel. We acquired samples in LSR Fortessa (BD). The percentage of positive EVs for each fluorochrome was determined using the fluorescence minus one (FMO) method. The mean fluorescence intensity (MFI) was determined in all EV regions. All analyses were performed using FlowJo 7.6.1 software.

### Western Blotting

A Western blot analysis was performed on plasma EVs from CNT, SAD, and FAD patients. The EVs were lysed in RIPA lysis buffer containing a protease and phosphatase inhibitor cocktail (Cytoskeleton, Biochem). The protein concentration was determined using a Pierce BCA protein assay kit (Thermo Fisher Scientific; Cat. #23225). BCA protein quantities were used for WB, transcytosis and LC-MS/MS analyses. Samples containing 50 μg of EV proteins were prepared in loading buffer (0.375 M TRIS, pH 6.8, 50% glycerol, 10% SDS, 0.5 M DTT and 0.002% bromophenol blue) and boiled at 95°C for 5 min. Afterward, the samples were resolved by electrophoresis at 120 V for 2 h on 12% sodium dodecyl sulfate–polyacrylamide gels and then transferred at 300 mA for 1 h to polyvinylidene difluoride (PVDF) membranes (BIO-RAD). Subsequently, the membranes were blocked with 5% non-fat milk in Tris-Buffered Saline (TBS; 20 mM Tris-HCl pH 7.5, 500 mM NaCl, pH 7.4) for 1 h at room temperature and incubated overnight at 4°C with a primary mouse antibody against claudin 5 (Invitrogen; Cat. #35-2500; 1:500) and a primary mouse antibody against Flotilin-1 (BD, Biosciences; Cat. 610821), both diluted in 5% BSA (Sigma-Aldrich, Cat. # A9647) and 0.2% Tween 20 in TBS. The membranes were repeatedly washed with TBS-T before 1 h of incubation with a goat anti-mouse IRDye 800CW secondary antibody (LI-COR Biosciences; Cat. #926-32210; 1:7000) at room temperature. An Odyssey Infrared Imaging System (LI-COR Biosciences) was used to visualize and quantify the signals scanned at 800 nm at a resolution of 169 μm.

### LC-MS/MS Analysis

Samples (25 μg) were loaded on an 8% precast RunBlue bis tris gel (Expedeon) and were run briefly at 100 V for 5 min. Gels were stained with Instant Blue Protein Stain (Expedeon) and subsequently washed with ultrapure water. Gel bands were excised and further minced into small pieces and completely de-stained using 70% 50 mM NH_4_HCO_3_ and 30% acetonitrile (ACN). Reduction and alkylation of cysteines was performed by the addition of 10 mM DTT dissolved in 50 mM NH_4_HCO_3_ and incubated at 56°C for 30 min. Next, 55 mM iodoacetamide in 50 mM NH_4_HCO_3_ was added and incubated with the samples for 30 min in the dark at room temperature. The remaining fluid was removed and 100% ACN was added after which the samples were incubated for 15 min. The supernatant was removed and the gel pieces were dried for 15 min at 56°C. Proteins were digested upon addition of 10 ng/μL sequencing-grade modified trypsin (Promega) in 50 mM NH_4_HCO_3_ to the gel pieces after which the samples were incubated overnight at 37°C. The following day, peptides were extracted using 5% formic acid followed by a second elution in 5% formic acid in 66% acetonitrile. Combined extracts were dried in a SpeedVac and dissolved in 20 μL 5% formic acid. LC-MS/MS of the tryptic peptides was performed using the Ultimate 3000 HPLC system coupled online to a Q-Exactive-Plus mass spectrometer with a NanoFlex source (both from Thermo Fisher Scientific) equipped with a stainless-steel emitter as previously described (Nagy et al., 2019). For the database search and label-free quantitation (LFQ), the software PEAKS X (Bioinformatics Solutions Inc., Waterloo, Ontario, Canada) was applied to the spectra to search against the Human SwissProt database (20,404 entries). The false discovery rate was set at 1% and at least one unique peptide should be present. LFQ was performed using the PeaksQ module incorporated into the PEAKS X software. Protein profile heat map was represented as a 1.2-fold change for each protein. Volcano plots showing significance vs. fold change for different combinations of experimental groups were also included. The *p*-values were obtained from a one-way analysis of variance (ANOVA) test. Both axes are plotted in the log scale. Boundaries for a *p*-value of 0.05 and fold changes of two times the magnitude of the mean between the groups under evaluation are also included as dotted gray lines.

Proteins were clustered in categories (biological function, Molecular Function, Protein Class and Pathway) using two different bioinformatics resources: DAVID Bioinformatics 6.8 (Huang et al., 2009a,b) and PANTHER 15 (Protein ANalysis THrough Evolutionary Relationships) database (Mi and Thomas, 2009; Mi et al., 2019). The whole human genome was employed as background list. The analysis of biological function was performed in DAVID selecting the GO terms for “Combined View for Functional Annotation.” Molecular Function, Protein Class and Pathway analysis were performed with DAVID bioinformatics resource. This analysis was coupled to the pathway enrichment analysis performed with PANTHER using the PANTHER Pathway keywords and exported as bar chart of number of genes.

The mass spectrometry proteomics data have been deposited to the ProteomeXchange Consortium via the PRIDE (Perez-Riverol et al., 2019) partner repository with the dataset identifier PXD021718.

### hCMEC/D3 Cell Culture

hCMEC/D3 cells were cultured in 25 cm^2^ culture flasks precoated with 150 μg/mL rat tail collagen type-I from Enzo Life Sciences (#ALX-522-435) in supplemented endothelial basal medium 2 (EBM-2, #CC-3156, Lonza). The following supplements were added to the EBM-2 at the indicated final concentrations: 5% (v/v) FBS, 10 mmol/L HEPES (#15630106, Gibco), 1% (v/v) chemically defined lipid concentrate (#11905-031, Gibco), 1.4 μmol/L hydrocortisone (#H0888, Sigma), 5 μg/mL ascorbic acid (#A4544, Sigma-Aldrich), 1 ng/mL basic fibroblast growth factor (#100-18D, PeproTech) and 1% (v/v) penicillin-streptomycin. Cells were passaged every 3 days at a split ratio of 1:10, and the medium was changed after 2 days.

### Transcytosis Assay

For the microvasculature transcytosis model, cells were seeded on 150 μg/mL collagen-coated polycarbonate membranes (0.4 μm pore size) in 12 well Transwell^TM^ plate inserts (#3401, Costar) at a density of 50,000 cells per cm^2^ and grown to confluency for 5 days. The medium was changed every 2 days with 0.5 mL and 1.5 mL medium added to the apical and basolateral compartments, respectively (De Jong et al., 2018). On day 5, the medium was removed from the apical and basolateral compartments, which were washed once with HBSS. EVs were stained with 1,1′-dioctadecyl-3,3,3′,3′-tetramethylindocarbocyanine perchlorate (DiI, Invitrogen). DiI was added to 100 μg of EVs at a final concentration of 1 μM after which the EVs were immediately vortexed. Next, the samples were centrifuged at 45,000 rpm in a TLA 100.3 rotor for 70 min. Protein content was quantified using a bicinchoninic acid (BCA) assay (BioRad), in which BSA (Sigma-Aldrich) was used as a standard. Then, 10 μg of EVs were apically added to the hCMEC/D3 cell monolayer, which was incubated for 16 h at 37°C in a humidified atmosphere of 5% CO_2_. The media from the apical and basolateral compartments were collected, and the apical and basolateral fractions were transferred into black flat-bottomed microplates (Greiner Bio-One 655209); their fluorescence intensities were quantified using a Fluostar-Optima microplate reader (BMG Labtech). After subtracting the respective background fluorescence, the percentage of fluorescence that was associated with the apical and basolateral fractions was calculated relative to the total fluorescent content, which accounted for the apical, basolateral and fluorescence intensities. Fluorescence was measured with an excitation filter (544–10 nm) and an emission filter (575–10 nm) using a Fluostar-Optima microplate reader (BMG Labtech). The percentage of transcytosis was calculated as follows:% Transcytosis = ([1.5 × F_basolateral]/[(0.6 or 0.5 × F_apical) + (1.5 × F_basolateral)]) × 100; where F_apical and F_basolateral are the mean fluorescence intensities of the apical and basolateral compartments, respectively.

### Primary Neuronal Cultures

Cortical samples from Wistar rat embryos (E18–19) were dissected and dissociated, and 5 × 10^4^ neurons were cultured on 12 mm coverslips precoated with poly-L-lysine (Sigma-Aldrich) in Neurobasal medium (GIBCO), which contained B-27 supplement (Sigma-Aldrich), L-glutamine and a penicillin-streptomycin antibiotic mixture (GIBCO). The samples were cultured at 37°C in a humidified atmosphere containing 5% CO_2_ (Posada-Duque et al., 2015a). After 14 days *in vitro* (DIV), neurons were stimulated with EVs for 24 h.

### Primary Astrocyte Culture

Cortical samples from postnatal day 1–2 Wistar rat pups were enzymatically dissociated with trypsin, cultured in 75-cm flasks at 37°C and 5% CO_2_, and maintained in DMEM (Sigma-Aldrich) supplemented with 10% fetal bovine serum (FBS, GIBCO) (Becerra-Calixto et al., 2018). The medium was changed every 2 days. The astrocyte-enriched cultures were obtained beginning at DIV 8 by shaking the flasks at 350 rpm for 48 h at 37°C; the culture medium was replaced every 48 h. Either live DIV 15 astrocytes or thawed cryopreserved DIV 15 astrocytes were plated onto 12 mm coverslips at a density of 4 × 10^4^ cells per coverslip. After 2 days, astrocytes were stimulated with EVs for 24 h.

### bEnd.3 Cell Line Culture

The bEnd.3 (ATCC CRL-2299) *Mus musculus* cell line was used as an endothelial cell model. bEnd.3 cells express von Willebrand factor and take up fluorescently labeled low-density lipoprotein (LDL) (Montesano et al., 1990). The bEnd.3 cells were thawed in DMEM supplemented with 20% FBS, a penicillin–streptomycin mixture, and 0.25% L-glutamine and were centrifuged at 1500 rpm for 5 min. The bEnd.3 cells were maintained in DMEM supplemented with 10% FBS, a penicillin-streptomycin mixture and L-glutamine and were incubated at 37°C in 5% CO_2_. bEnd.3 cells were trypsinized in a 0.25% trypsin/EDTA mixture for 5 min and subcultured into 12 mm coverslips precoated with gelatin at a density of 2.5 × 10^5^ cells per coverslip. When a 100% confluent monolayer was observed, bEnd.3 cells were stimulated with EVs for 24 h.

### bEnd.3 Cell and Astrocyte Cocultures

bEnd.3 cells were subcultured on previously gelatinized coverslips containing paraffin spots (spot size was approximately 0.5 mm high and 2 mm wide). In parallel, primary astrocytes were subcultured in 12-well plates (Becerra-Calixto et al., 2018). On DIV16, the bEnd.3 cells and primary astrocytes were cocultured for 4 days. On DIV 20 for astrocytes, cocultures were stimulated with EVs for 24 h.

### Neuron and Astrocyte Cocultures

Neurons were isolated as mentioned above and cultured on poly-lysine-coated coverslips with paraffin “feet” (paraffin dots adhered to coverslips, approximately 0.5 mm high and 2 mm wide) (Posada-Duque et al., 2015b). At DIV 8 for neurons, DIV 15 astrocytes were thawed in coverslips. At DIV 10 for neurons, the coverslips containing the neurons were moved to the astrocyte plates, suspended on the astrocytes, and maintained in serum-free neurobasal medium for 7 days. At DIV 17 for neurons, the cocultures were treated with EVs for 24 h.

### Brain Cortical Organoids

To obtain mature brain cortical organoids, we followed a previously published protocol (Pasca et al., 2015). We cultured embryonic stem cells (Hues 9, obtained from the Harvard Stem Cell Core Facility) on hESC- qualified Matrigel (Corning) in mTeSR1 medium (STEM CELL Technologies) supplemented with 0.2% Mycozap plus-PR (Lonza) at 37°C with 5% CO_2_. We then generated cortical bodies by seeding the cells in an AggreWell 800 ultra-low attachment plate (10,000 cells per well, STEM CELL Technologies) with 10 μM of the ROCK inhibitor Y27632 (Bio-Connect) for 24 h. We transferred the cortical bodies to ultra-low attachment T75 flasks (Corning). The cortical organoids were maintained in DMEM/F12 supplemented with 20% KnockOut Serum Replacement, 0.5% glutamax, 1% penicillin–streptomycin (Gibco), 1 mM non-essential amino acids (Thermo Fisher Scientific), 0.1 mM 2-mercaptoethanol (Sigma-Aldrich) and SMAD inhibitors (10 μM SB-431542 purchased from Tocris and 5 μM Dorsomorphin purchased from Sigma-Aldrich) from days 0–6; the medium was changed daily. From days 6–25, we cultured the organoids in neurobasal A medium (Gibco) with B27 without vitamin A, 1% glutamax, 1% penicillin–streptomycin and 0.1% Mycozap plus-PR supplemented with 20 ng/ml EGF (Sigma-Aldrich) and FGF (PeproTech); the medium was changed every 2 days after day 16. On day 26, for further maturation, the medium was supplemented with 20 ng/ml NT-3 and BDNF (PeproTech) in place of EGF and FGF. After day 43, we maintained the organoids in the same medium but without NT-3 and BDNF. On day 90, we stimulated the cortical organoids with EVs for 48 h.

### Preparation and Stimulation With EVs

We stimulated all the above cell cultures and brain cortical organoids with CNT-, SAD-, and FAD-EVs at a cell:EV ratio of 1:1. EVs were thawed at room temperature and washed with 1× PBS at 16,900 × *g* for 1 h, and then, determined amounts of EVs were added at a 1:1 ratio. For 3-month-old organoids, the cell number estimate was determined according to an approximate of cell proliferation at day 90 and following a previous published protocol that indicated cell proliferation in the brain organoids (Pasca et al., 2015). Cell cultures were stimulated for 24 h and brain cortical organoids were stimulated for 48 h with EVs resuspended in fresh medium. As a control, we added fresh medium without EVs. All the stimulations were performed in medium depleted of EVs by filtration through a 0.1 μM membrane.

### Cell Death Analysis by LDH Release Measurement

We assessed cell death by measuring the lactate dehydrogenase (LDH) released from the cultures using a cytotoxicity detection kit (Roche). We recovered the culture supernatant after EV treatment. We determined LDH activity by measuring the NADH absorption using a spectrophotometer to determine the linear rate of NADH consumption during the reduction of pyruvate to lactate. The percentage of cytotoxicity was calculated for a given test condition using the following equation: cytotoxicity (%) = [(*A* − low control)/(high control − low control)] × 100, where *A* represents the mean LDH activity in the media from three wells per duplicate of the given test conditions, low control represents LDH release from the untreated control cells, and high control represents the maximal LDH release from the cells (treated with 1% Triton X-100 for 24 h for cells, and 4% Triton X-100 for 24 h for organoids).

### Cell Microscopy Labeling Immunofluorescence

For the morphological and phenotypical analyses, we fixed and stained astrocyte-endothelium and astrocyte-neuron cocultures. Initially, we fixed the cell cultures in 4% paraformaldehyde prepared with cytoskeleton buffer for 20 min. Autofluorescence was eliminated by incubating the cultures in 50 mM NH_4_Cl for 10 min. We permeabilized the cells and blocked non-specific protein interactions with PBS + 0.2% Triton X-100 for 5 min and PBS + 2.5% FBS for 1 h. We incubated the cells with mouse primary antibodies against GFAP (Sigma, 1:1000) and p120 catenin (Sigma, 1:1000) overnight at 4°C, followed by incubation with Alexa Fluor^®^ 488 or 594 secondary antibodies (Molecular Probes, 1:1000). Nuclei were stained with Hoechst 33,258 (Invitrogen, 1:5000), and phalloidin conjugated to Alexa Fluor^®^ 594 or Alexa Fluor^®^ 488 (Molecular probes, 1:2000) was added for 1 h to stain nuclei and F-actin, respectively. We washed the cells four times with PBS, cover-slipped them with FluorSave^TM^ and observed them under an Olympus IX 81 epifluorescence microscope. No staining was observed when the primary antibodies were omitted. We captured six to eight images per coverslip with at least two coverslips per condition.

### Morphometric Analysis

We performed image analysis for the astrocyte-endothelium and astrocyte-neuron cocultures. The sample size was defined as each patient (*n*). We reported all measurements as the fold change. Fold changes were calculated by dividing the values obtained from cells after treatment with CNT-EVs, SAD-EVs or FAD-EVs by the value obtained from cells with no EV treatment. We measured the mean fluorescence intensity (MFI) of GFAP per field (20×, 12 fields per *n*) in astrocytes from both coculture conditions, and we measured the MFI of p120 catenin per field (20×, 12 fields per *n*) in the endothelium. MFI was obtained by measuring the mean gray value of each field. In addition, we measured the “gap” area fraction per field and the gap size in endothelia (Becerra-Calixto et al., 2018). For this measurement, we binarized F-actin images using the same threshold for all conditions. The masked areas were considered gaps. We measured the area fraction per field (20×, 12 fields per *n*). Within the masked area, we calculated the gap size using particle size analysis (20×, 12 fields per *n*). We used a similar strategy in neurons, and binarized F-actin images using the same threshold for all conditions. The masked area was considered F-actin-positive. We then measured the area fraction per field (20×, 12 fields per *n*).

### Calcium Live-Organoid Imaging

For organoid calcium imaging, we used 90-day organoids and stimulated them with mixes of EVs (pool) from three individuals belonging to the control, SAD and FAD groups. After 48 h, organoids were incubated with Fluo4-Direct according to the manufacturer’s instructions (Invitrogen) and imaged using a DeltaVision microscope (Environmental chamber, 37°C and CO_2_ control; UPFLN 40× oil, NA 1.3, WD 0.2 mm). We recorded five fields per organoid, and frames were captured every 20 s over 30 min. The initial 15 min allowed capture of the basal signal, while the 15 min after 200 μM glutamate treatment were also captured. Glutamate was prepared in Fluo4-Direct solution (1 M HEPES, 1× HBSS, pH 7.3). We calculated Δ*F*/*F*_0_ from the mean gray value (MFI) from the overall fields. Δ*F*/*F*_0_ was described as: Δ*F* = (*F*_*frame*_ − *F*_*lowest value*_); *F*_0_ = mean Δ*F* of the first five frames.

### Statistical Analysis

The sample size (*n*) of each experimental group was defined as each patient and analyzed as an independent assay. For the data acquired, we tested normality using the Shapiro–Wilk and Kolmogorov tests. We analyzed parametric univariable data using one-way ANOVA, followed by one-way ANOVA with Tukey’s multiple comparison test. We analyzed non-parametric data using the Kruskal–Wallis test and Dunn’s multiple comparison test. For the multivariable analysis, we used two-way ANOVA, followed by the Bonferroni *post hoc* test for comparisons between several independent groups. We processed all groups in parallel to reduce the interassay variation. The data are expressed as means plus the SEM or as medians plus interquartile ranges depending on the normality of the data analyzed. The analyses were performed using PRISM software. The results were considered significant at ^∗^ indicates *p* < 0.05; ^∗∗^ indicates *p* < 0.01, ^∗∗∗^ indicates *p* < 0.001and ^****^ indicates *p* < 0.0001.

Additionally, multivariate statistical analyses were performed using a partial least squares-discriminant analysis (PLS-DA) (Liu and Rayens, 2007). The PLS-DA was included because it is particularly suitable for the analysis of datasets with a small number of samples and a large number of variables. The PLS-DA analysis was performed according to a previously described process (Ballabio and Consonni, 2013). The variable importance in projection (VIP) index [Mehmood] was estimated. The VIP is a weighted sum of the squares of the PLS weight that indicates the importance of each variable in the model and reflects the proportion of the explained variance weighted by the covariance between the predictor variables and dependent variables—i.e., the groups. For comparison, the significance multivariate correlation (sMC) [Tran] index was also included in the analyses. The sMC is similar to the VIP but discards residual variance in the predictor variables that can be considered non-relevant information to discriminate among the groups. The confidence ellipsoids per group were also included.

## Results

### CLN-5 Spots Are Increased Near the Surface of the Brain Microvasculature in AD Brains

Since CLN-5-positive EVs released by brain microvascular endothelial cells were suggested to be a mechanism for leukocyte transendothelial migration during neuroinflammation (Paul et al., 2016), we evaluated the presence of these CLN-5+ structures near the microvasculature in CNT, SAD and FAD human brains. Staining revealed a significant increase in microvascular CLN-5 both in SAD and FAD tissues compared with CNT tissues (Figures 1A,B and Supplementary Figure S1). Interestingly, an abundant microvascular dotted-like staining pattern (black arrowheads and insets) was observed in SAD and FAD, while CNT samples showed a subtle CLN-5 reactivity. To characterize this pattern, we labeled the microvascular basal lamina (UEA I+) and performed confocal imaging analysis restricted to these structures. Despite a broad and diffused CLN-5 labeling profile along the tissue, we observed particularly intense fluorescent signals from CLN-5+ spots inside and surrounding the microvessels in both CNT and AD tissues (Figures 1C,C’). Image analysis revealed a tenfold increase in the number of these CLN-5+ spots in SAD samples compared with CNT samples and around a fourfold rise comparing with FAD samples (Figure 1D), whereas FAD was not significantly different from CNT.

To assess whether these CLN-5+ spots were morphologically different among the experimental groups, we compared their mean volumes. However, it was found that the average volume of the CLN-5+ spots in the three conditions was similar and ranged from ∼0.017 to 0.022 μm^3^ (Figure 1E). Lastly, since we observed CLN-5 spots luminally (red-filled arrowheads; Figures 1C,C’), abluminally (white-filled arrowheads) and at different distances away from the microvessel surfaces (empty arrowheads), we determined the percentage of total CLN-5+ spots present inside the microvasculature and the percentage distribution of these particles through an arbitrary range of 5 μm from the abluminal microvessel surface (Figure 1F). Representative 3D reconstructions of the microvessels and the spots are shown in Figure 1G. In both CNT and AD tissues, most of the total CLN-5+ spots present near the microvessels correspond to luminal particles (>62%; Figure 1F, left panel). Moreover, SAD showed 28% more luminal CLN-5+ spots than CNT and 15% more than FAD. Regarding the abluminal distribution of CLN-5+ spots, SAD tissue exhibited a greater percentage of particles apposed directly on the vessel surface (particles present between 0 to 1 μm from the vessel surface) compared with CNT tissue (>99% in SAD vs. ∼86% in CNT) (Figure 1F, right panel); the SAD tissue also contained slightly wider distribution of CLN-5+ spots throughout the entire evaluated distance. Although the distribution of CLN-5+ spots in FAD was similar to that in SAD (>94% particles close to the surface), the difference compared with the CNT group was not statistically significant. Together, our results showed that CLN-5+ spots are associated with the brain microvasculature and are increased in SAD.

### EVs Are Increased in AD Patients

**FIGURE 2:**
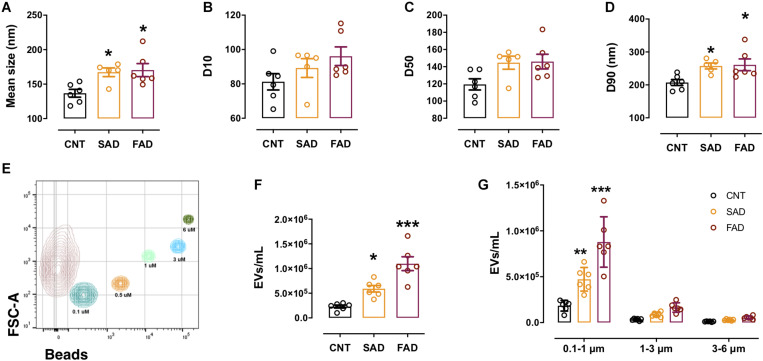
The concentration and number of EVs are increased in the plasma of AD patients. **(A)** Mean size of EVs as measured by NTA from CNT (black), SAD (orange), and FAD (red) patients. **(B)** D10, **(C)** D50, and **(D)** D90 parameters obtained by NTA. **(E)** Representative contour plot of a control sample EV population and reference beads of different sizes. **(F)** Concentration of EVs/mL as measured by flow cytometry in the entire region and **(G)** in the different size intervals: MVs, 0.1–1; apoptotic bodies, 1–6. For **(A–D)**, the representative data from CNT, *n* = 6; SAD, *n* = 5; FAD, *n* = 6. For panels **(E–G)**, representative data from CNT, *n* = 6; SAD, *n* = 6; FAD, *n* = 6. Data are plotted as means and SEM. One-way ANOVA, Tukey’s multiple comparison test. * indicates *p* < 0.05; *** indicates *p* < 0.001.

Based on the finding that CLN-5+ spots were increased in the microvasculature of the AD brain, we isolated systemic EVs and found that CLN-5 and Flotillin-1 were part of the molecular composition in these EVs (Supplementary Figure S2A). Next, to characterize systemic-EVs, we compared the size distribution and concentration of CNT-, SAD-, and FAD-EVs. Individual NTA histograms are shown in Supplementary Figure S2B. We detected particles ranging from 50 to 700 nm in size, which correspond to the sizes of exosomes and MVs. No differences in concentration were found among the samples (Supplementary Figure S2C). We found a significant increase in the mean size of SAD- (mean = 173.4 nm) and FAD-EVs (mean = 178.3 nm) compared with CNT-EVs (mean = 143.8) (Figure 2A). D10 shows the lower limit of 10% of the measured particles, D50 shows half of the measured particles (50%) and D90 shows the upper limit of 90% of the measured particles. No difference was observed in D10 and D50 among the samples (Figures 2B,C). In contrast, we found a significant increase in D90 in the SAD- and FAD-EVs compared with the CNT-EVs (Figure 2D).

Using flow-cytometry, we detected particles ranging from 100 to 6,000 nm in size, which corresponded to the sizes of MVs and apoptotic bodies (ABs) (Figure 2E). We found a significant increase in the concentration of SAD- and FAD-EVs compared with CNT-EVs (Figure 2F). Furthermore, we used size reference beads to elucidate in which size interval the EVs differ. The size intervals were: 0.1–1, 1–3, and 3–6 μm. We found a significant increase in EV concentration only in the 0.1–1 μm region, which corresponds to MVs (Figure 2G). We found a lower concentration of MVs and ABs in CSF compared with plasma (Supplementary Figure S2F), but we did not find a difference in CSF-EVs among the different groups (Supplementary Figure S2G). These results suggest that systemic EVs are increased in AD.

### Differential Phenotype of Systemic EVs in FAD and SAD

**FIGURE 3:**
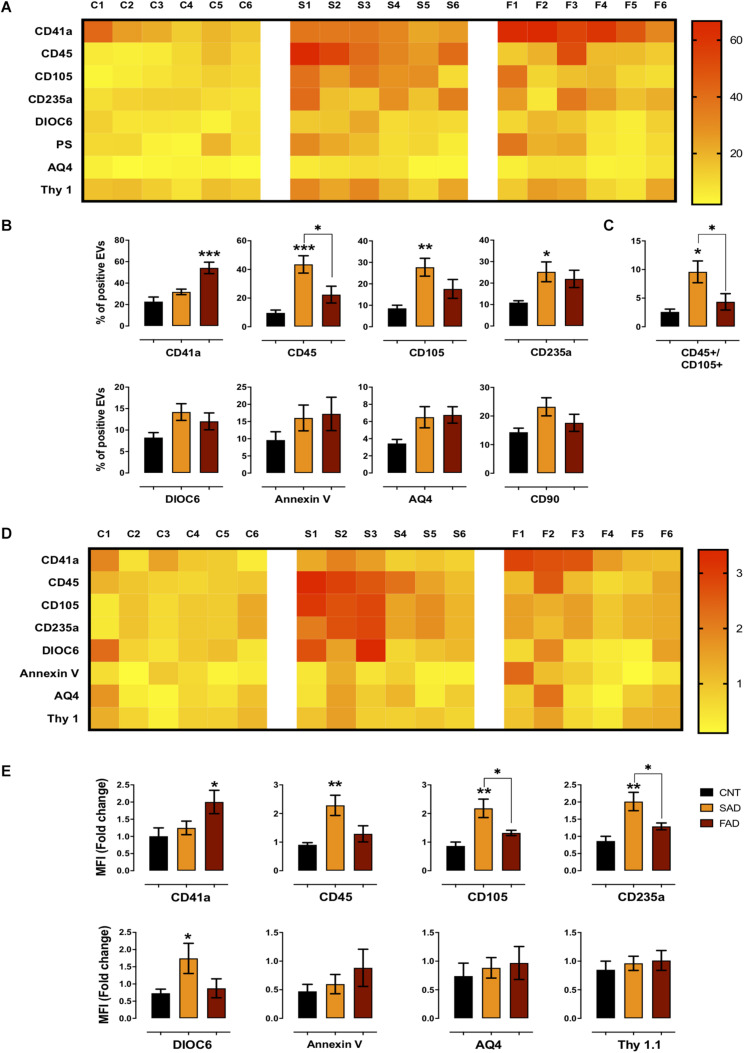
Differential phenotype of SAD- and FAD-EVs. **(A)** Heatmap of the percentages of EVs positive for cell markers (CD41, platelets; CD45, leukocytes; CD105, endothelia; CD235a, erythrocytes; AQ4, astrocytes; CD90, neuronal-like; Annexin, phosphatidylserine and DIOC6, mitochondria) from each CNT, FAD and SAD sample. **(B)** Comparison of the percentages of EVs positive for cell markers in the grouped data in panel **(A)**. **(C)** Percentages of EVs double-positive for markers (CD45, leukocyte; CD105, endothelium) in CNT, FAD and SAD. **(D)** Heatmap of the normalized MFI of cell markers (to mean data from CNT individuals). **(E)** Comparison of the normalized MFI of cell markers for the grouped data in panel **(C)**. Representative data from CNT, *n* = 6; SAD, *n* = 6; FAD, *n* = 6. Data are plotted as means and SEM. One-way ANOVA, Tukey’s multiple comparison test.; * indicates *p* < 0.05; ** indicates *p* < 0.01; *** indicates *p* < 0.001.

To determine the phenotype of systemic-EVs, we analyzed blood and brain cell markers, mitochondria and PS exposure using flow cytometry (gating strategy according to that shown in Supplementary Figure S3). As a visual representation of the percentage of positive events and MFI, two heatmaps are shown with six samples per group (Figures 3A,D). Only SAD showed an increased percentage of EVs positive for leukocyte (CD45+), endothelial (CD105+) and erythrocyte (CD234a) markers (Figures 3A,B); this increase was also seen in the MFI for leukocyte and erythrocyte markers and for mitochondria (DIOC6) in SAD-EVs compared with CNT-EVs (Figures 3D,E). Interestingly, only FAD had an increased percentage of positive EVs and MFI for a platelet marker (CD41a+) (Figures 3A,B,D,E). Additionally, SAD-EVs exhibited increased MFI for endothelial and erythrocyte markers (Figures 3D,E) and had EVs that were double-positive for leukocyte and endothelial markers (CD45+/CD105+) compared with FAD-EVs (Figure 3C). Moreover, AD-EVs exhibited a tendency to have an increased percentage of EVs positive for mitochondria, astrocytes (AQ4) and neuronal-like (CD90) markers compared with control EVs. These results suggest that SAD-EVs have an endothelial-leukocyte phenotype and that FAD-EVs have a platelet phenotype.

### Differential Quantities of Protein Composition in SAD- and FAD-EVs

**FIGURE 4:**
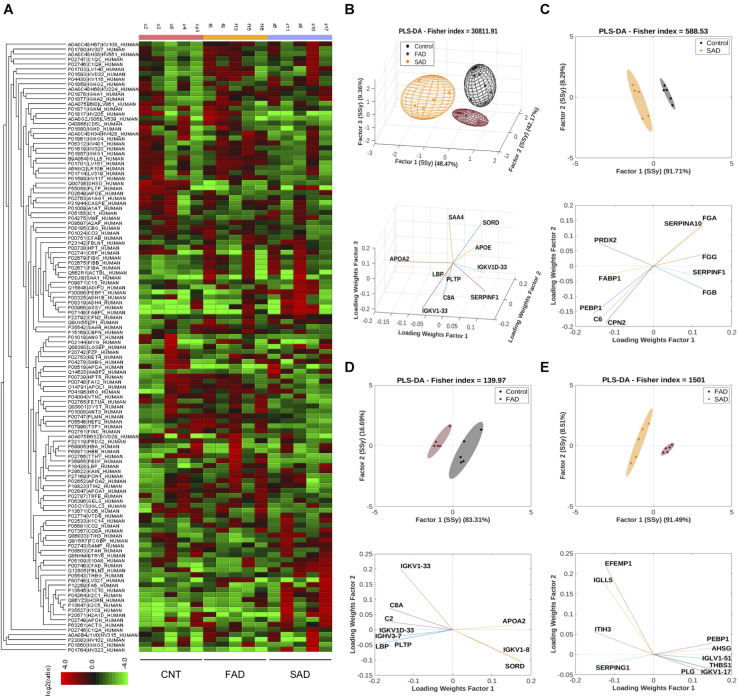
Protein compositions of SAD- and FAD-EVs. **(A)** Heatmap of the protein fold changes for each sample compared with the mean of CNT-EVs. In all, 130 proteins were considered by using a 1.2-fold change and an FDR ≤ 1%. **(B)** Multivariable analyses from CNT-, SAD-, and FAD-EVs. PLS-DA was used to discriminate between protein abundance in CNT compared with SAD **(C)**, CNT compared with FAD **(D)**, and SAD compared with FAD **(E)**. For panels **(A–E)**, representative data from CNT, *n* = 5; SAD, *n* = 5; FAD, *n* = 5.

To determine the protein composition of EVs, we used an LC-MS label-free approach and analyzed samples from CNT, FAD, and SAD patients. In all, 130 proteins were identified (FDR 0.1%, Supplementary Table S1) with a differential abundance for each sample. The protein profile heatmap of the 130 proteins is shown (Figure 4A), where each color represents the log2(ratio) of the average abundance across different samples normalized to the CNT-EV average. Hierarchical clustering was generated using a neighbor-joining algorithm with a Euclidean distance similarity measurement of the log2 of the protein ratios of each sample (for one group) or each group (for multiple groups). The PLS-DA analysis indicated a protein profile for all experimental groups, which were distinguished by three factors (Figure 4B and Supplementary Table S2). The proteins SORD, SAA4, LBP, SERPINF1, PLPT, APOE, APOA2, IGKV1D-33, and IGKV1-33, C8A were used to distinguish the groups. In addition, the PLS-DA analysis indicated that the control group was located in a differential spatial plane than the FAD (Figure 4C) and SAD (Figure 4D) groups. The fibrinogen gamma, alpha and beta chain (FGG, FGA, FGB) proteins, complement C6, peroxiredoxin 2 (PRDX2), phosphatidylethanolamine-binding protein 1 (PEBP1), carboxypeptidase N subunit 2 (CPN2), pigment epithelium-derived factor (SERPINF1), fatty acid binding protein 1 (FABP1), and serine protease inhibitor 10 (SERPINA 10) were used to distinguish the SAD group from the CNT group (Figure 4C). Complement C2 and C8A, SORD, PLPT, immunoglobulins (IGKV1D-33, IGKV1-33, IGVH3-7 and IGVK1-8), LBP, and APOA2 were used to distinguish the FAD group from the CNT group (Figure 4D). Additionally, the PLS-DA analysis indicated that FAD was located in a different spatial plane than SAD (Figure 4D). Inter-alpha-trypsin inhibitor heavy chain H3 (ITIH3), alpha-2-HS-glycoprotein (AHSG), thrombospondin-1 (THBS1) PEBP1, EGF-containing fibulin-like extracellular matrix protein (EFEMP1), immunoglobulin lambda like polypeptide 5 (IGLL5), plasmin (PLG), plasma protease C1 inhibitor (SERPING1) and immunoglobulins (IGKV1-17 IGLV1-51) could also be used to discriminate among these groups (Figure 4E).

**FIGURE 5:**
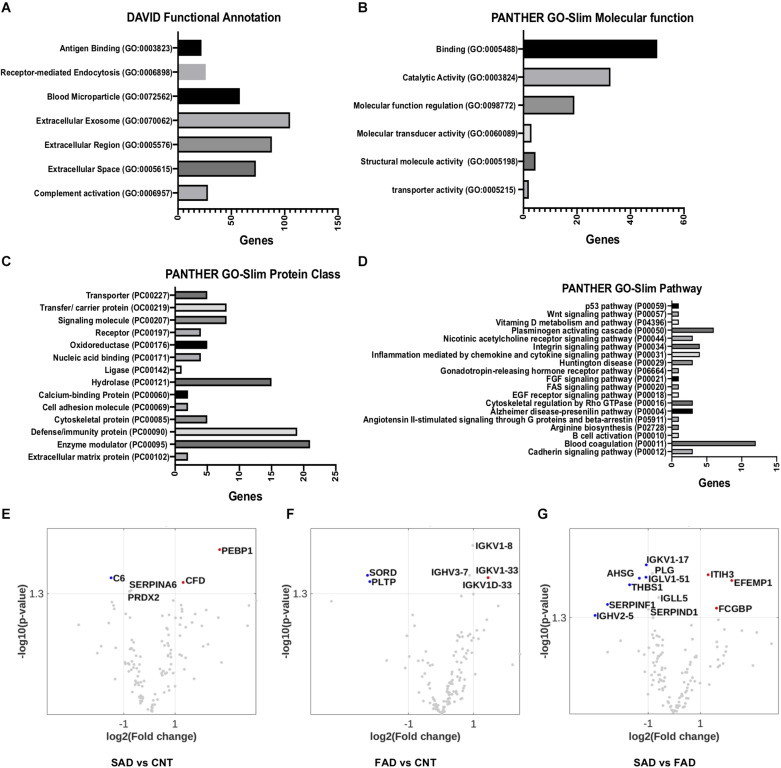
Pathways involved in EV protein composition and differential protein composition abundance in SAD- and FAD-EVs. **(A)** Functional Annotation based on DAVID, **(B)** Molecular Function, **(C)** Protein Class and **(D)** Signaling Pathway based on the PANTHER GO-Slim annotation of 130 proteins analyzed in EVs. Volcano plot showing the proteins that are upregulated or downregulated in **(E)** SAD-EVs compared with CNT-EVs, **(F)** in FAD-EVs compared with CNT-EVs **(G)** and in SAD-EVs compared with FAD-EVs. Names are shown for proteins with a log2-fold change higher than 1. For panels **(A–E)**, representative data from CNT, *n* = 5; SAD, *n* = 5; FAD, *n* = 5.

Using DAVID bioinformatics for functional GO terms we observed proteins from: “Extracellular Exosome,” “Extracellular Region,” “Extracellular Space,” “Blood Microparticle,” “Receptor-Mediated Endocytosis,” “Complement Activation,” and “Antigen Binding” (Figure 5A). Using PANTHER GO-Slim, the following GO molecular function terms were included in the analysis: “Binding,” “Catalytic Activity,” and “Molecular Function Regulator” were the main functions associated with the proteins detected in EVs (Figure 5B). Additionally, GO protein class terms such as “Enzyme modulator,” “Defense/Immunity Protein,” and “Hydrolase,” and the GO pathway terms “Blood Coagulation,” “Plasminogen Activity Pathway,” and “Inflammation Mediated by Chemokine and Cytokine Signaling Pathway” were the main components associated with the proteins detected in EVs (Figures 5C,D).

Volcano plots show the differentially expressed proteins in the different samples. Proteins are shown as a log2-fold change higher than 1 and with a significance of *p* < 0.05 (Figures 5E–G and Supplementary Table S2). SORD and PLTP (phospholipid transfer protein) were decreased in FAD compared with CNT; complement C6 was decreased in SAD compared with CNT; and SERPINF1, THBS1, AHSG and immunoglobulins were decreased in SAD compared with FAD. Immunoglobulins were increased in FAD compared with CNT; PEBP1 and complement Factor D (CFD) were increased in SAD compared with CNT; and IgGFc-binding protein (FGCBP), ITH3 and EFEMP1 were increased in SAD compared with FAD. Together, our results suggest a differential protein composition of EVs from SAD and FAD, which may be related to the coagulation cascade, inflammation and lipid-carbohydrate metabolism.

### AD-EVs Cross the Human Microvasculature Cells and Induce Endothelial Disruption and Astrocyte Hyperreactivity

**FIGURE 6:**
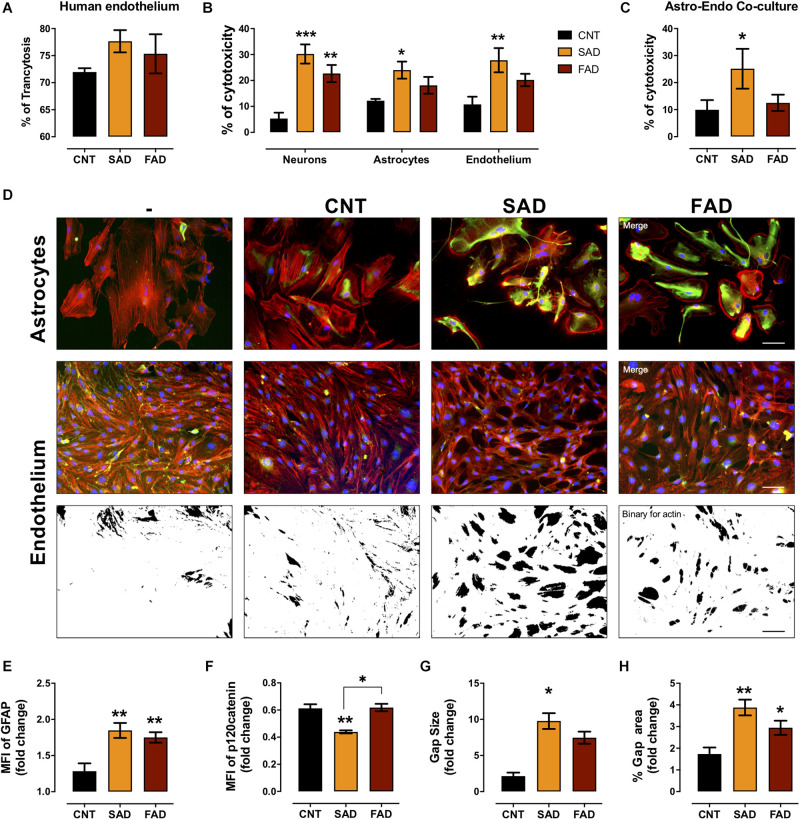
AD-EVs cross the BBB and induce endothelial dysfunction and astrocyte hyperactivity. **(A)** Percentage of transcytosis of CNT-, SAD-, and FAD-EVs through hCMEC/D3 human brain microvasculature cells. **(B)** The cytotoxicity in neuronal, astrocytic and endothelial monocultures, and **(C)** the cytotoxicity in astrocyte-endothelial cocultures expressed as the percentage of LDH released from cells 24 h after CNT-, SAD-, FAD-EVs treatment. **(D)** Morphological characterization of astrocytes: F-actin is shown in red, the GFAP cytoskeleton is shown in green and nuclei are shown in blue. Magnification 20×, scale bar 100 μm. Endothelium: F-actin is shown in red, p120 catenin is shown in green and nuclei are shown in blue. Magnification 20×, scale bar 100 μm. Fold change of GFAP **(E)** and p120 catenin **(F)** intensity (MFI) in astrocytes and endothelium treated with CNT-, SAD-, FAD-EVs, respectively. Fold change of the gap size **(G)** and the percentage of the gap area **(H)** of the endothelium treated with CNT-, SAD-, FAD-EVs. For panel **(A)**, representative data from CNT, *n* = 3; SAD, *n* = 3; FAD, *n* = 3. For panels **(B–H)**, representative data from CNT, *n* = 5; SAD, *n* = 5; FAD, *n* = 5. Data are plotted as means and SEM. One-way ANOVA, Tukey’s multiple comparison test. * indicates *p* < 0.05; ** indicates *p* < 0.01; *** indicates *p* < 0.001.

To assess whether systemic-EVs cross the microvasculature and interact with cells of the brain parenchyma, we performed a transcytosis assay using hCMEC/D3 human microvasculature cells. More than 70% of CNT-, SAD-, and FAD-EVs cross the microvasculature (Figure 6A). After, monocultures and cocultures of NVU cells were treated with EVs. Although the percentage of cytotoxicity induced was similar between SAD-EVs and FAD-EVs in monocultures, only SAD-EVs induced a significant increase in astrocyte and endothelial cytotoxicity

(Figure 6B). Moreover, in the endothelium-astrocyte coculture, only SAD-EVs caused an increase in cytotoxicity (Figure 6C).

To assess the cellular effect induced by EVs in cocultures, we analyzed astrocyte- and endothelial-specific markers (Figure 6D). Despite that both SAD- and FAD-EVs increased the MFI of glial fibrillary acid protein (GFAP) in astrocytes and the gap area in between endothelial cells (Figure 6E), only SAD-EVs decreased the MFI of the adherent junction protein p120 catenin (Figure 6F) and increased the size of gaps within the endothelium (Figures 6G,H). These results show that both types of AD-EVs cross the BBB and induce endothelial disruption and astrocyte hyperreactivity, while these effects are enhanced by SAD-EVs.

### AD-EVs Increase Neurotoxicity and Induce Calcium Dysregulation in Human Brain Organoids

**FIGURE 7:**
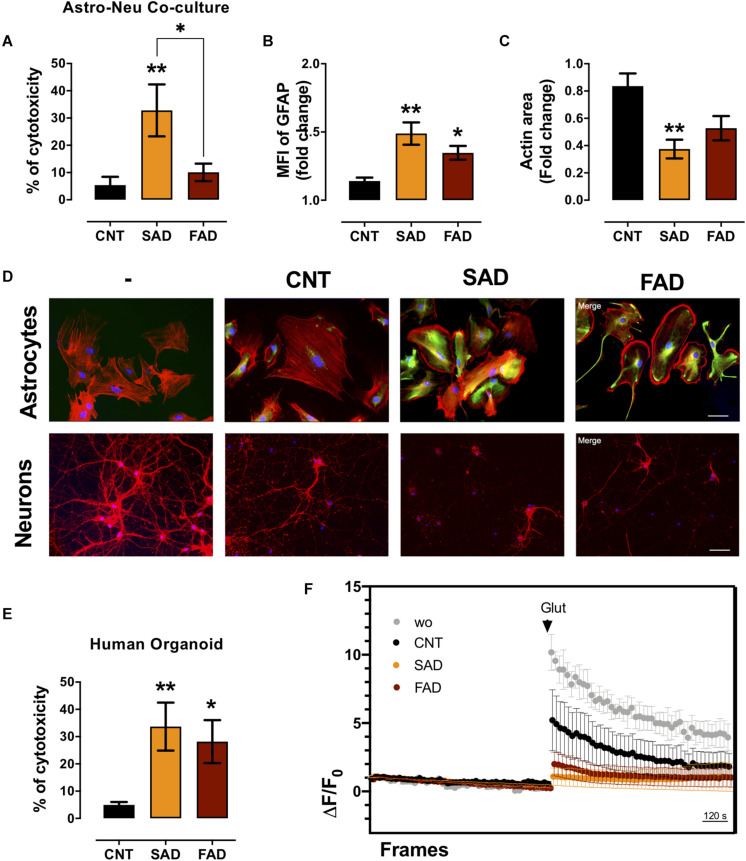
AD-EVs induce neurotoxicity, astrocyte hyperreactivity and calcium dysregulation. **(A)** The cytotoxicity of astrocyte-neuron cocultures is expressed as the percentage of LDH released from cells 24 h after CNT-, SAD-, FAD-EV treatment. **(B,C)** Fold change data were calculated by dividing the values by the value obtained from cells with no EV treatment. **(B)** Fold change of the GFAP intensity (MFI) of astrocytes treated with CNT-, SAD-, FAD-EVs. **(C)** Fold change of the percentages of F-actin per field of neurons treated with CNT-, SAD-, FAD-EVs. **(D)** Morphological characterization showing the following: Astrocytes: F-actin is shown in red, the GFAP cytoskeleton is shown in green and nuclei are shown in blue. Magnification 20×, scale bar 100 μm. Neurons: F-actin is shown in red, and nuclei were stained with Hoechst (blue). Magnification 20×, scale bar 100 μm. **(E)** The cytotoxicity of human organoids is expressed as the percentage of LDH released from cells 48 h after CNT-, SAD-, FAD-EV treatment. **(F)** Human cortical organoids treated with CNT-, SAD-, FAD-EVs for 48 h were incubated with Fluo4 (cytoplasmic calcium) and were imaged every 20 s for a total of 30 min (15 min to measure baseline activity and 15 min after the addition of 200 μM glutamate). The Δ*F*/*F*o fluorescence ratio was quantified in five fields. For panel **(A–F)**, representative data from CNT, *n* = 5; SAD, *n* = 5; FAD, *n* = 5. In panel **(E)**, representative data from CNT, *n* = 6; SAD, *n* = 5; FAD, *n* = 6. In panel **(F)**, the data represent a pool of EVs from CNT, *n* = 3; SAD, *n* = 3; FAD, *n* = 3. Data are plotted as means and SEM. One-way ANOVA, Tukey’s multiple comparison test. * indicates *p* < 0.05; ** indicates *p* < 0.01.

Since AD-EVs induced astrocyte-endothelial activation and disruption, we tested the effect of systemic-EVs on neuroglial components. Neuronal and astrocytic mono- and cocultures were treated with SAD- and FAD-EVs. Both types of AD-EVs induced neuronal cytotoxicity in monocultures; however, only SAD-EVs caused increased LDH release in astrocytic-neuronal cocultures (Figures 6B, 7A). These effects were related to astrocytic hyperactivity, as shown by an increase in GFAP, and diminished neuronal branches area, as shown by a decrease in F-actin area (Figures 7B–D).

To further understand the implication of systemic-EVs on a 3D culture model, we treated 90-day-old human cortical brain organoids. Interestingly, both SAD- and FAD-EVs caused increased cytotoxicity (Figure 7E). Furthermore, as a functional experiment, we measured calcium regulation (Δ*F*/*F*0) after stimulation with EVs. We recorded the basal calcium signal and changes in the signal after glutamate addition. Interestingly, a whole-field analysis of calcium change (Δ*F*/*F*0) showed that EVs reduced the calcium response after glutamate addition, but this reduction was more drastic in response to both FAD- and SAD-EVs (Figure 7F). The data suggest that both types of AD-EVs induce cytotoxicity and impair calcium dynamics after glutamate exposure in human cortical organoids.

## Discussion

Here, we characterized AD-EVs from SAD and FAD patients and determined their effect on NVU cells and human cortical organoids. In AD, we observed an increase in brain microvascular and systemic MVs, and the cellular origin of systemic EVs was associated with leukocyte-endothelium in SAD patients and platelets in FAD patients. Using proteomics, we detected a differential protein composition of SAD- and FAD-EVs. The protein components were implicated in the coagulation cascade, inflammation and lipid-carbohydrate metabolism. Specifically, PEBP1, CFD, and C6 represented the protein signature of SAD-EVs, while SORD, PLPT, and IGGKV1D-33 represented that of FAD-EVs. We also observed that both types of AD-EVs induced processes associated with AD pathophysiology, in particular, BBB disruption, astrocyte hyperactivation, neuronal death and calcium dysregulation. Endothelial detrimental effects generated by SAD-EVs were higher than those generated by FAD-EVs. We propose that differential EV phenotypes in the sporadic and familiar forms of AD are associated with neuroglial and endothelial degeneration. To our knowledge, this is the first study in which human cortical organoids were stimulated with EVs and the first to suggest that PEBP1 and fibrinogen in systemic EVs of SAD patients could be mediators of neurovascular dysfunction. This finding suggests that these proteins may serve as prodromal biomarkers that could be used to clarify some early events in the pathogenesis of AD and as a basis for a directed future preventive treatment.

Growing evidence indicates that EVs are important in a variety of neurodegenerative and neuroinflammatory diseases (Malm et al., 2016; Li T.R. et al., 2019) including AD (Joshi et al., 2014; Laske et al., 2015; Hosseinzadeh et al., 2018; Yang et al., 2018; Cha et al., 2019; Kapogiannis et al., 2019; Pulliam et al., 2019). We detected an increased size and number of systemic EVs in SAD and FAD, which could be related to an increase in EV generation pathways (budding/exocytosis), alterations in vesicle trafficking or a deficiency in EV clearance (exo/endocytosis). We detected an increase in larger EVs, which may correspond to microvesicles (MVs). Increased MVs production has been proposed as a response to chronic inflammation (Słomka et al., 2018), and in AD, an increase in the size and number of systemic EVs has been previously reported (Kapogiannis et al., 2019). The *PS1* mutation in FAD alters vesicle trafficking (Zhang et al., 2006; Stenovec et al., 2016); moreover, the *E280A* mutation is associated with the *EXOC2* mutation, which alters exocytosis/endocytosis pathways (Vélez et al., 2016). In SAD, the *ApoE4* (Konttinen et al., 2019a) and *TREM2* (Kleinberger et al., 2014) mutations have been associated with reduced phagocytosis by immune cells, which could be involved in the EV increase seen in AD. Additionally, a recent study found altered EV biogenesis and autophagy impairment (failure to include LAMP-1 in lysosomes) in preclinical models and clinical AD patients (Gallart-Palau et al., 2020), which could be associated with the EV-brain size and count increase seen in AD. Autophagy markers should therefore be further evaluated in systemic EVs to better clarify this association.

Systemic EVs can be generated from different cell sources, such as platelets, leukocytes, erythrocytes and endothelial cells (Akbar et al., 2019). We showed that the phenotype of SAD-EVs was derived from endothelium and leukocytes and that they exhibited increased mitochondrial marker expression. An increase in endothelial-EVs has been linked to injured microvasculature (Andrews et al., 2016; Ramirez et al., 2018), and this increase has been previously reported in AD patients with vascular dysfunction (Hosseinzadeh et al., 2018). An increase in leukocyte-EVs has been proposed to be associated with leukocyte activation (Pugholm et al., 2016). In AD, activation of neutrophils (Dong et al., 2018), monocytes (Fani Maleki and Rivest, 2019) (cells that phagocytose amyloid-β) (Zuroff et al., 2017), and T (Gate et al., 2020) and B lymphocytes (Söllvander et al., 2015; Pluta et al., 2018) has been proposed (Pugholm et al., 2016). Moreover, in SAD-EVs, the increased expression of the mitochondrial marker DIOC6 could be associated with increased mitochondria in EVs as a bioenergetic compensatory mechanism (Torralba et al., 2016) or it could indicate dysfunctional mitochondria (Konttinen et al., 2019b; Picca et al., 2019). This result support our previous finding and show a differential influence on mitochondria in SAD through a significant increase in the phospholipid PE-CER compared with FAD (Villamil-Ortiz et al., 2018). Furthermore, mitophagy attenuates disease impairment in a mouse model of the disease (Fang et al., 2019).

Endothelial-EVs have been associated with leukocyte infiltration (Ramirez et al., 2018), specifically the generation of CLN-5+ endothelial-leukocyte complexes (Paul et al., 2016). We observed CLN-5 sphere pattern-like spots, which may be EVs, but this still remain to be verified by ultrastructural analysis using Electron Microscopy (EM) or CryoEM. In both types of AD-EVs, we detected increased CLN-5 spots closer to the microvasculature in the brain parenchyma, and endothelial and leukocyte EVs, which could be associated with immune infiltration and higher neurovascular impairment in SAD. Interestingly, in both AD forms, we found a tendency for astrocytes and neuronal-like EVs to increase, which could be the result of astrocyte hyperactivity (Chun and Lee, 2018) and neurovascular degeneration in AD. Additionally, we found that the phenotype in FAD samples with the E280A PS1 mutation was related to platelets. Activated platelets have been described in AD; these platelets retain more amyloid-β and amyloid-β precursor protein (Davies et al., 1997), and platelet granules are systemic reservoirs of presenelin-1 (Mirnics et al., 2002), which could explain the FAD-E280A platelet phenotype of EVs.

Using an unbiased LC-MS label-free approach, we observed an increase in size of EVs from AD patients and differential protein composition. Interestingly, systemic AD-EVs have altered expression of lipid metabolism proteins (ApoE, ApoA1, PLTP, PEBP1). Lipid metabolism has been implied in neurological and psychiatric diseases (Liu et al., 2015), specifically AD (Kalvodova et al., 2005; Hirsch-Reinshagen et al., 2009; Villamil-Ortiz et al., 2018). In addition, our group found that the phospholipid profile had a common disbalance in the frontal cortex and white matter in dementias such as AD and CADASIL, which may be related to changes in neurotransmission, microgliosis and NVU impairment (Sabogal-Guáqueta et al., 2020). Interestingly, the phospholipid profile of the temporal cortex showed a differential content of PE (phosphatidylethanolamine) in SAD and FAD patients (Villamil-Ortiz et al., 2018). This content may be associated with the current finding that systemic SAD-EVs have altered PEBP1, which we propose could be related to the disbalance in mitochondrial metabolism (Calzada et al., 2016). This disturbance is related to the metabolic alterations that have been described in AD (Van Der Velpen et al., 2019). Even so, this finding implies that the dysregulation of glucose metabolism, as was suggested by our results, SORD could distinguish AD with respect to CNT-EVs and FAD to CNT-EVs. SORD is associated with an alternate route of glucose metabolism, and this pathway is believed to be involved in the diabetic neuropathy linked to AD (Dewanjee et al., 2018).

Complement components and proinflammatory cytokines have been detected in EVs (Goetzl et al., 2018; Yang et al., 2018) and are related to platelet activation and an altered coagulation process (De Luca et al., 2018). Both types of AD-EVs have altered contents of complement cascade proteins, such as C1q, C2, CFD, C8A and immunoglobulins. High complement levels have been shown in astrocyte-EVs from AD patients (Goetzl et al., 2018). Complement has been associated with neuroinflammation in AD, where dysregulation of the neuroimmune system leads to complement overactivation, glial activation and neuronal death (Morgan, 2018). Moreover, in SAD, polymorphisms in complement receptor 1 *(CR1)* have been associated with the disease (Harold et al., 2009). Antibodies against amyloid β, which have been reported in AD patients (Toro et al., 1999), could drive the activation of the classical component cascade. Interestingly, SAD-EVs have an altered content of the coagulation protein fibrinogen, which is related to vascular dementia and AD (Sun et al., 2020). Moreover, plasma fibrinogen levels have been found to be increased in AD, and polymorphisms in the fibrinogen regulatory gene are linked to AD (Sun et al., 2020). Fibrinogen has been proposed to play a role in NVU degeneration (Li Y. et al., 2019), and fibrinogen plaques have been detected in AD brains (Cortes-Canteli et al., 2015). Additionally, serum amyloid can bind to fibrin or fibrinogen and increase plaque formation (Page et al., 2019).

Multiple studies have shown that EVs can cross the BBB (García-Romero et al., 2017; Matsumoto et al., 2017) and we also showed that AD-EVs can cross the BBB (in an *in vitro* model of brain microvasculature cells) and interact with NVU cells. We propose that EVs bind to NVU cells and deliver enzymes associated with lipids, metabolism, complement and the coagulation process, which induce cytotoxicity, cellular activation and functional dysregulation (calcium dynamics) in NVU components. In addition, protein aggregates (serum amyloid) or prion-like proteins (Pérez et al., 2019), proteases (calpain) (Laske et al., 2015), and cytokines (You et al., 2020) could be associated with this effect. Interestingly, EVs associated with Aβ can mediate calcium dysregulation in AD models (Eitan et al., 2016). SAD-EVs induced higher cytotoxicity and cellular activation, which correlate with the vascular damage seen in late-onset AD (Iturria-Medina et al., 2016). In contrast, FAD-EVs are higher in number, which could also explain their detrimental effects. Nevertheless, in human cortical organoids, both types of AD-EVs induced cytotoxicity and impaired calcium dynamics in response to glutamate, demonstrating the importance of both types of EVs in neuroglial degeneration. Here, we propose that the calcium response to glutamate is impaired due to cell death and circuit loss in cortical organoids. Additionally, we found CNT-EVs halved calcium response to glutamate, which may be explained by a basal cytotoxic response against plasma EVs. It may be related to CNT-EVs crossing of the BBB in the *in vitro* model, and a partial increased in GFAP expression in astrocytes, gaps formation in the endothelium and decreased neuronal coverage compared to medium control conditions. However, an altered level of calcium has been proposed in AD and is related to cell death (Berridge, 2011).

Despite, limited access to human cells may present translational limitations when rodent cells are stimulated with human samples, in this study we confirmed the effects induced by systemic-EVs in rodent NVU cells in cortical organoids. Also, our *in vitro* model does not mimic the complex integrity and functionality of the NVU, therefore additional *in vivo* studies should be performed to better understand the effect of these systemic-EVs in the NVU. Also, access to AD postmortem samples limit additional clinical analysis such as age and sex implication in AD EVs. Furthermore, postmortem patients have comorbidities that may influence our analysis. Despite SAD patients had comorbidities related to hypertension, cardiovascular disease and dyslipidemia, which we related to systemic and metabolic disorder, CNT also had hypertension and malignancy as main comorbidities, which we relate to age. Therefore, we cannot exclude that SAD EVs effects in the evaluated patients could be influenced by the mentioned comorbidities. Additionally, state of the art EV-isolation based on size exclusion as a chromatography or immune-bead isolation could determine which type and role of EVs (exosome, microvesicles or apoptotic bodies) mostly implicated in AD (Théry et al., 2018); and whether the EVs-depletion experiments prevents NVU cell damage.

In summary, differential EV phenotypes in the sporadic and familiar forms of AD are associated with neuroglial and endothelial degeneration. We propose that AD-EVs could reflect alterations in neuroimmune homeostasis, which are characterized by an increased EV count, size, and differential protein composition related to the coagulation cascade, complement activation, and lipid metabolism regulators. Our results suggest that AD-EVs can contribute to the disease as mediators of NVU degeneration. Above all, SAD-EVs are specifically related to vascular alterations associated with late-onset AD pathogenesis. As a limitation of our study we worked with postmortem samples, so we propose a validation of EVs in life cohort patients. Further studies should be performed to elucidate the role of EVs as biomarkers for AD diagnosis so that pharmacological interventions can be designed for AD treatment.

## Data Availability Statement

The datasets presented in this study can be found in online repositories. The names of the repository/repositories and accession number(s) can be found below: https://www.ebi.ac.uk/pride/archive/, PXD021718.

## Ethics Statement

The studies involving human participants were reviewed and approved by Comité de Bioética de Investigación en Humanos, SIU. Record 17-10-755. The patients/participants provided their written informed consent to participate in this study. The animal study was reviewed and approved by Comité de Ética para la Experimentación con Animales de la Universidad de Antioquia (CEEA). Record 110, May 17th 2017. Written informed consent was obtained from the individual(s) for the publication of any potentially identifiable images or data included in this article.

## Author Contributions

JV-V performed experiments and analyses of research. JV-V, JH-R, LB, AV, GC-G, and RP-D designed the experiments. JH-R performed human tissue experiments and analyses. AV and DA sampling and diagnosis of human tissue. DC designed flow cytometer experiments. LB and DV performed organoid model. LB performed proteomic experiments. LR and IZ performed transcytosis and nanotracking experiments and analyses. JA-L performed proteomic multivariable analyses. JV-V, JH-R, GC-G, and RP-D analyzed the data. JV-V, JH-R, and RP-D wrote the manuscript. JV-V, JH-R, LB, AV, DC, JA-L, IZ, DV, LR, GC-G, and RP-D reviewed and edited the manuscript. All authors contributed to the article and approved the submitted version.

## Conflict of Interest

The authors declare that the research was conducted in the absence of any commercial or financial relationships that could be construed as a potential conflict of interest.

## Supplementary Material

Supplementary Figure 1 Increased CLN-5 microvascular staining in AD. **(A)** Low magnification brain images of four different CNT, SAD, and FAD tissues showing increased IHC for CLN-5 in the entire sampled area of all AD cases compared with CNT, in which a very scarce and subtle reactivity was observed. **(B)** High magnifications of the previous images showing that most of the microvessels are CLN-5-positive in both SAD and FAD tissues. Moreover, two different staining patterns were identified: microvascular dotted-like (black-filled arrowheads) and parenchymal spot-like (empty arrowheads) staining patterns. A detailed examination of the CNT images showed that although most of the CLN-5 staining is restricted to the microvasculature, many of the microvessel-like structures appear CLN-5-negative (red-filled arrowheads). Scale bars: **(A)**, 500 μm; **(B)**, 50 μm.

Supplementary Figure 2 CLN-5 and Flotillin-1 Western blot, NTA analysis and EVs in plasma and CSF. **(A)** Western blot results of CLN-5 and Flotillin-1 in CNT-EVs (C1–C3), SAD-EVs (S1–S3) and FAD-EVs (F1–F3). **(B)** Concentration vs. size NTA histograms of CNT-EVs (C1–C5), SAD-EVs (S1–S5) and FAD-EVs (F1–F5). **(C)** Concentration (EVs/mL) according to NTA. **(D)** Comparison of the concentration of CSF- and plasma-EVs. **(E)** Concentration of CSF-EVs from CNT, SAD and FAD tissues. In panel **(A)**, representative data from CNT, *n* = 3; SAD, *n* = 3; FAD, *n* = 3. For panels **(B,C)**, representative data from CNT, *n* = 5; SAD, *n* = 5; FAD, *n* = 6. In panel **(D)**, plasma EVs from *n* = 6; SAD, *n* = 6; FAD, *n* = 6. For panels **(D,E)**, CSF EVs from *n* = 4; SAD, *n* = 5; FAD, *n* = 5. Data are plotted as means and SEM. Mann–Whitney test. ^∗∗∗^ indicates *p* < 0.01.

Supplementary Figure 3 Representative flow cytometry gating strategy of cell markers for Figure 3. Positive events were established according to fluorescence minus one (FMO) in CNT-, SAD-, and FAD-EVs. Representative contour plot for CD41a, CD45, and CD105. Representative dot plot for CD235a, DIOC6, Annexin V, CD90, and AQ4. Representative data from CNT, *n* = 6; SAD, *n* = 6; FAD, *n* = 6.

Supplementary Figure 4 Dynamic cytosolic calcium in organoids treated with glutamate. Representative surface profile of Fluo-4 from organoids incubated with CNT, SAD-, and FAD-EVs at baseline, after glutamate addition and during the final condition.

Supplementary Table 1 Proteins detected by the LC/MS proteomic approach in the analyzed samples. Peak output data of 130 proteins detected in the analyzed samples. Protein group, protein ID, accession, significance, coverage (%), #peptides, #unique, PTM, average, mass, description.

Supplementary Table 2 Multivariant PLS-DA analysis index. VIP, sMC, and SR index for 130 proteins compared between groups (CNT vs. SAD and FAD), (CNT vs. SAD), (CNT vs. FAD), (SAD vs. FAD).
