# Supplementary figures and images for: Differential Profile of Systemic Extracellular Vesicles From Sporadic and Familial Alzheimer’s Disease Leads to Neuroglial and Endothelial Cell Degeneration
Source: Front Aging Neurosci. 2020 Nov 11;12:587989. doi: 10.3389/fnagi.2020.587989 (PMC7705379; doi:10.3389/fnagi.2020.587989)

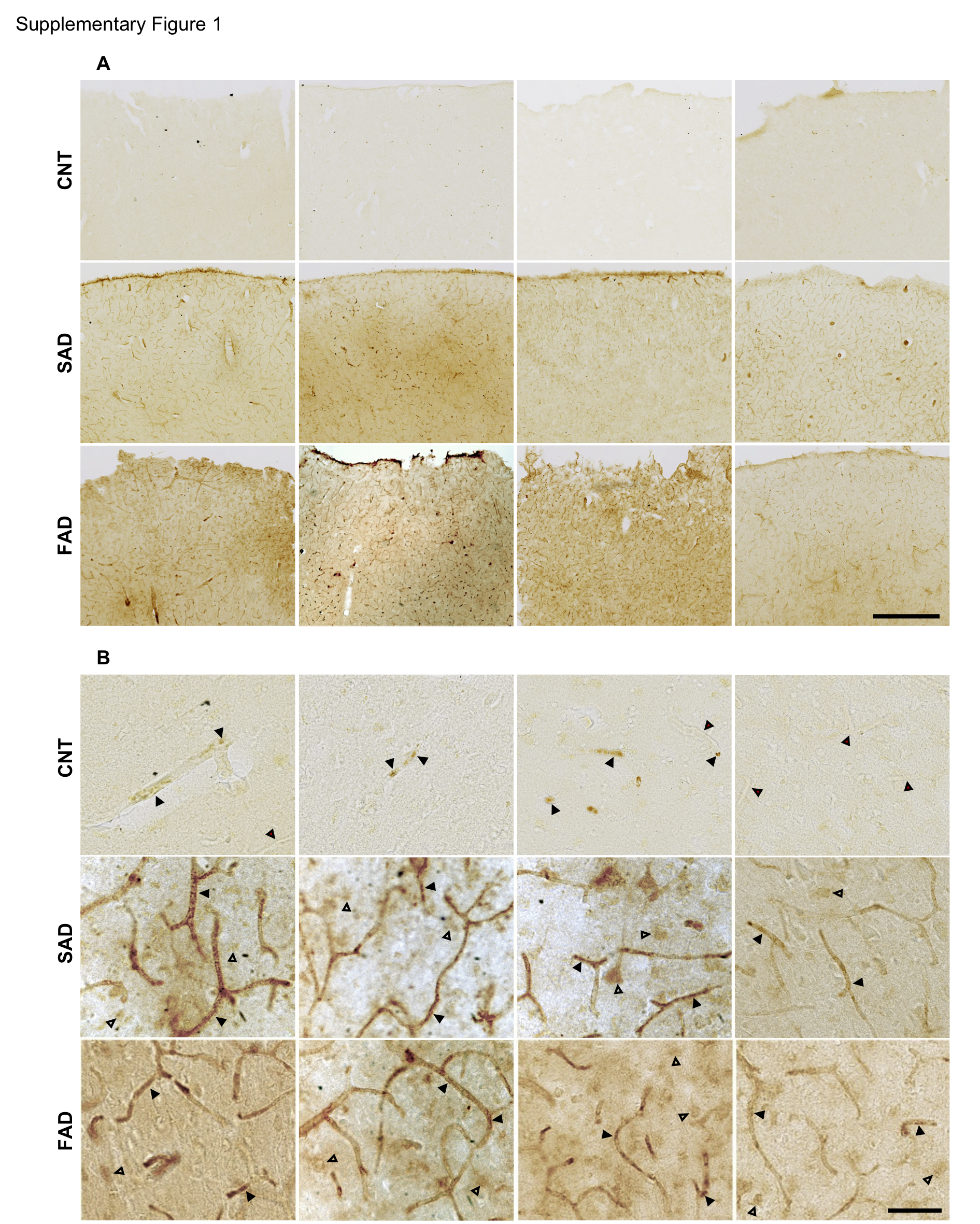

Supplement: Supplementary Figure 1 — Increased CLN-5 microvascular staining in AD. (A) Low magnification brain images of four different CNT, SAD, and FAD tissues showing increased IHC for CLN-5 in the entire sampled area of all AD cases compared with CNT, in which a very scarce and subtle reactivity was observed. (B) High magnifications of the previous images showing that most of the microvessels are CLN-5-positive in both SAD and FAD tissues. Moreover, two different staining patterns were identified: microvascular dotted-like (black-filled arrowheads) and parenchymal spot-like (empty arrowheads) staining patterns. A detailed examination of the CNT images showed that although most of the CLN-5 staining is restricted to the microvasculature, many of the microvessel-like structures appear CLN-5-negative (red-filled arrowheads). Scale bars: (A), 500 μm; (B), 50 μm. [file Image_1.TIFF]

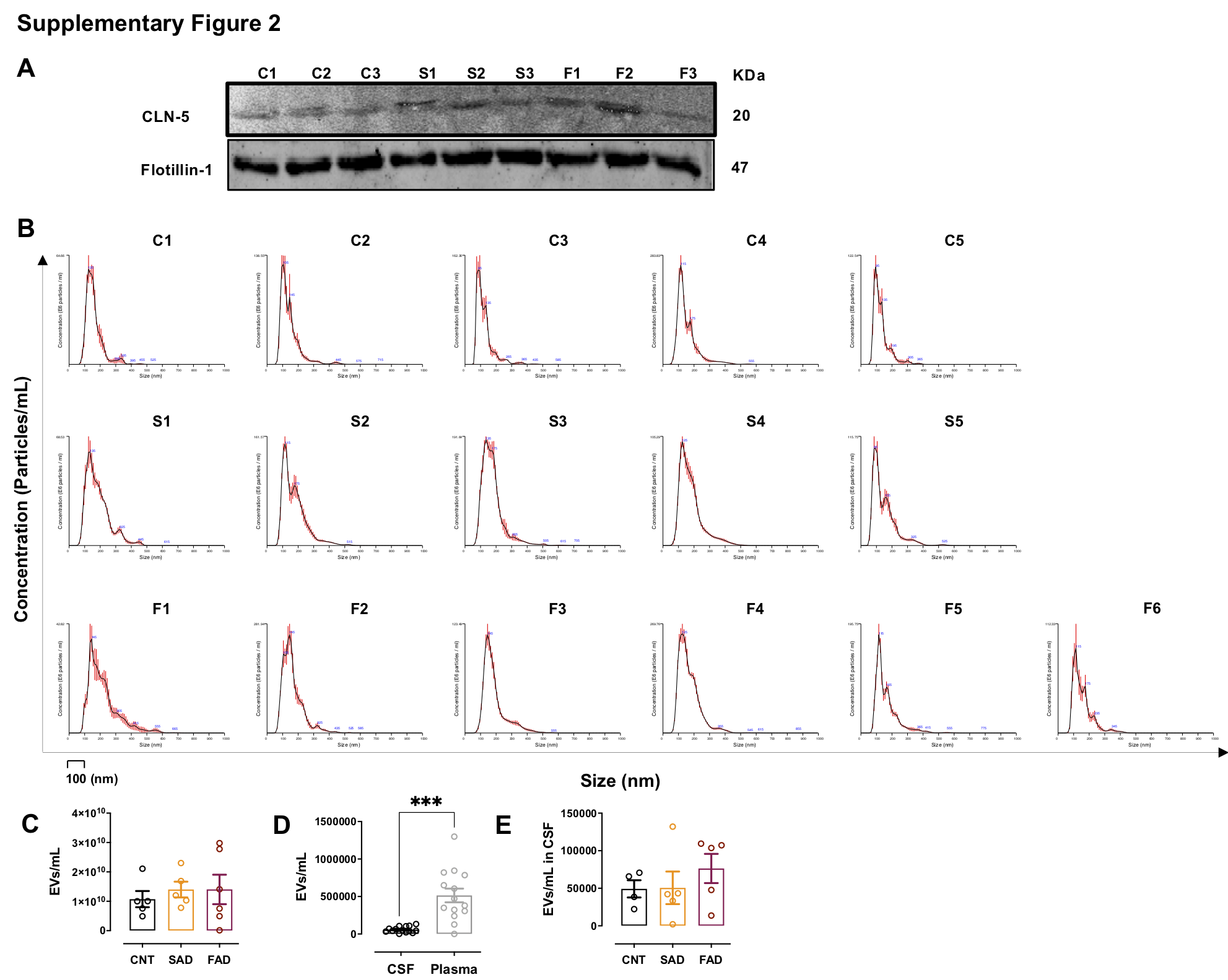

Supplement: Supplementary Figure 2 — CLN-5 and Flotillin-1 Western blot, NTA analysis and EVs in plasma and CSF. (A) Western blot results of CLN-5 and Flotillin-1 in CNT-EVs (C1–C3), SAD-EVs (S1–S3) and FAD-EVs (F1–F3). (B) Concentration vs. size NTA histograms of CNT-EVs (C1–C5), SAD-EVs (S1–S5) and FAD-EVs (F1–F5). (C) Concentration (EVs/mL) according to NTA. (D) Comparison of the concentration of CSF- and plasma-EVs. (E) Concentration of CSF-EVs from CNT, SAD and FAD tissues. In panel (A), representative data from CNT, n = 3; SAD, n = 3; FAD, n = 3. For panels (B,C), representative data from CNT, n = 5; SAD, n = 5; FAD, n = 6. In panel (D), plasma EVs from n = 6; SAD, n = 6; FAD, n = 6. For panels (D,E), CSF EVs from n = 4; SAD, n = 5; FAD, n = 5. Data are plotted as means and SEM. Mann–Whitney test. ∗∗∗ indicates p < 0.01. [file Image_2.TIFF]

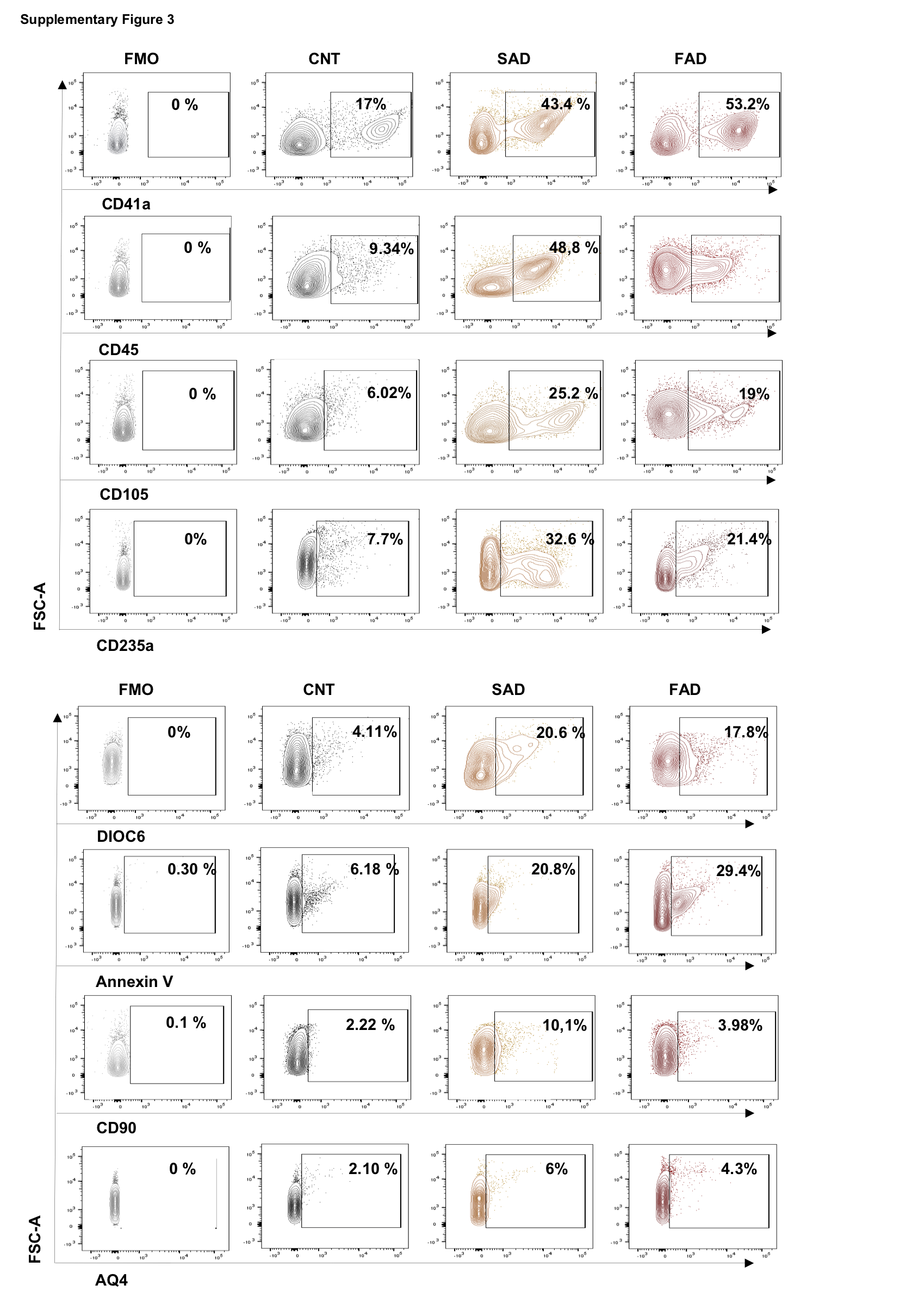

Supplement: Supplementary Figure 3 — Representative flow cytometry gating strategy of cell markers for Figure 3. Positive events were established according to fluorescence minus one (FMO) in CNT-, SAD-, and FAD-EVs. Representative contour plot for CD41a, CD45, and CD105. Representative dot plot for CD235a, DIOC6, Annexin V, CD90, and AQ4. Representative data from CNT, n = 6; SAD, n = 6; FAD, n = 6. [file Image_3.TIFF]

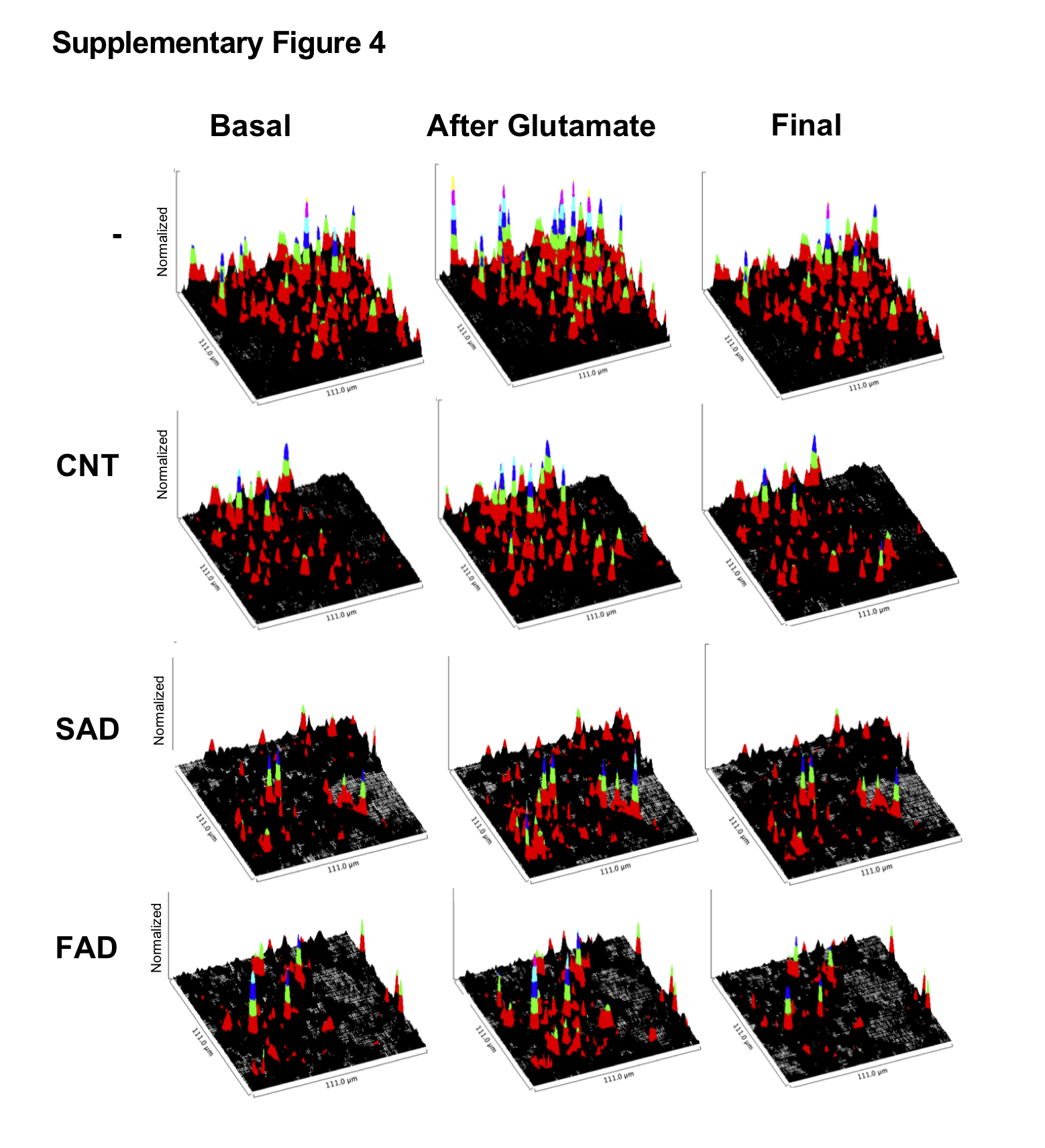

Supplement: Supplementary Figure 4 — Dynamic cytosolic calcium in organoids treated with glutamate. Representative surface profile of Fluo-4 from organoids incubated with CNT, SAD-, and FAD-EVs at baseline, after glutamate addition and during the final condition. [file Image_4.TIFF]
